# Metabolic and phylogenetic diversity in the phylum *Nitrospinota* revealed by comparative genome analyses

**DOI:** 10.1093/ismeco/ycad017

**Published:** 2024-01-10

**Authors:** Linnea F M Kop, Hanna Koch, Mike S M Jetten, Holger Daims, Sebastian Lücker

**Affiliations:** Department of Microbiology, Radboud Institute for Biological and Environmental Sciences, Radboud University, Heyendaalseweg 135, Nijmegen 6525 AJ, The Netherlands; Division of Microbial Ecology, Centre for Microbiology and Environmental Systems Science, University of Vienna, Djerassiplatz 1, Vienna 1030, Austria; Department of Microbiology, Radboud Institute for Biological and Environmental Sciences, Radboud University, Heyendaalseweg 135, Nijmegen 6525 AJ, The Netherlands; Bioresources Unit, Center for Health & Bioresources, AIT Austrian Institute of Technology GmbH, Konrad-Lorenz-Straße 24, Tulln an der Donau 3430, Austria; Department of Microbiology, Radboud Institute for Biological and Environmental Sciences, Radboud University, Heyendaalseweg 135, Nijmegen 6525 AJ, The Netherlands; Division of Microbial Ecology, Centre for Microbiology and Environmental Systems Science, University of Vienna, Djerassiplatz 1, Vienna 1030, Austria; Department of Microbiology, Radboud Institute for Biological and Environmental Sciences, Radboud University, Heyendaalseweg 135, Nijmegen 6525 AJ, The Netherlands

**Keywords:** Nitrospinota, metagenomics, nitrification, nitrite oxidation, sulfide oxidation

## Abstract

The most abundant known nitrite-oxidizing bacteria in the marine water column belong to the phylum *Nitrospinota*. Despite their importance in marine nitrogen cycling and primary production, there are only few cultured representatives that all belong to the class *Nitrospinia*. Moreover, although *Nitrospinota* were traditionally thought to be restricted to marine environments, metagenome-assembled genomes have also been recovered from groundwater. Over the recent years, metagenomic sequencing has led to the discovery of several novel classes of *Nitrospinota* (UBA9942, UBA7883, 2-12-FULL-45-22, JACRGO01, JADGAW01), which remain uncultivated and have not been analyzed in detail. Here, we analyzed a nonredundant set of 98 *Nitrospinota* genomes with focus on these understudied *Nitrospinota* classes and compared their metabolic profiles to get insights into their potential role in biogeochemical element cycling. Based on phylogenomic analysis and average amino acid identities, the highly diverse phylum *Nitrospinota* could be divided into at least 33 different genera, partly with quite distinct metabolic capacities. Our analysis shows that not all *Nitrospinota* are nitrite oxidizers and that members of this phylum have the genomic potential to use sulfide and hydrogen for energy conservation. This study expands our knowledge of the phylogeny and potential ecophysiology of the phylum *Nitrospinota* and offers new avenues for the isolation and cultivation of these elusive bacteria.

## Introduction

Nitrite-oxidizing bacteria (NOB) play a key role in the marine nitrogen cycle. Nitrate produced by nitrification is the main bioavailable form of nitrogen in the open ocean, which represents a growth-limiting factor for marine organisms [1, 2]. The known genera of NOB belong to four different phyla: the *Pseudomonadota* (formerly Proteobacteria), *Nitrospirota*, *Nitrospinota*, and *Chloroflexota* [3]. Of these, members of the phylum *Nitrospinota* are the most dominant known marine nitrite oxidizers in the water column, contributing up to 9% of the microbial community in oxygen minimum zones (OMZs) [1, 4–7]. *Nitrospinota* are not only the key nitrite oxidizers in the ocean [6, 8]; they also fix 15%–43% of inorganic carbon in the Northern Atlantic and thus have a significant impact on carbon cycling and primary production as well [6].

Even though *Nitrospinota* are phylogenetically diverse and play a key role in marine environments, they are notoriously recalcitrant to cultivation and only five cultured representatives that all are affiliated with the class *Nitrospinia* have been reported. All of these have been isolated or enriched from seawater or marine sediment samples [9–12]. Contrastingly, metagenomics has led to the discovery of novel yet uncultured *Nitrospinota* classes (UBA9942, UBA7883, 2-12-FULL-45-22, JACRGO01, JADGAW01) [13]. These *Nitrospinota* single amplified genomes and metagenome-assembled genomes (SAGs and MAGs, respectively) have been recovered from a wide range of habitats such as suboxic and open ocean waters, sponges, and (hydrothermal) sediments [5, 14–18]. Although it was generally assumed that *Nitrospinota* were restricted to marine habitats, they were recently detected in subsurface metagenomes as well [19–21]. However, most of these novel *Nitrospinota* MAGs have not yet been analyzed in detail.

The main distinguishing feature of NOB is their chemolithoautotrophic lifestyle using nitrite and CO_2_ as their sole energy and carbon sources, respectively [3]. Although a recent study concluded that the majority of NOB in the dark ocean (where no sunlight penetrates) rely on nitrite oxidation alone for energy conservation [6], there is evidence that some NOB are more versatile and not limited to nitrite as energy source [8, 22]. For example, *Nitrospira moscoviensis* of the phylum *Nitrospirota* can grow by aerobic hydrogen oxidation [22, 23] and can also oxidize formate, either aerobically or coupled to nitrate reduction under anoxic conditions using the nitrite oxidoreductase (NXR) in reverse [24]. Formate or acetate oxidation with nitrate reduction was also shown for the marine species *Nitrococcus mobilis* (phylum *Pseudomonadota*) [8]. Furthermore, *N. mobilis* is capable of sulfide oxidation for detoxification and might even be able to grow using sulfide as its energy source [8]. Although the cultured *Nitrospinota* species have limited metabolic versatility [12, 25, 26], several genome-based studies have suggested that members of this phylum could be involved in sulfur cycling and nitrogen fixation. MAGs belonging to the *Nitrospinota* class UBA7883 were found to encode reverse dissimilatory sulfite reductases that is involved in the oxidation of sulfide (DsrAB). Initially, these genes were identified in groundwater MAGs [27], but *dsrAB* genes were also found in a marine MAG belonging to this class [28]. Recently, the ancestral metabolic profile of the sister phyla *Nitrospirota* and *Nitrospinota* was reconstructed, suggesting that sulfur, hydrogen, and one-carbon-based metabolisms were metabolic traits of the common ancestor of these phyla [29]. Our study expands on these findings by reconstructing the metabolic potential of six classes within the phylum *Nitrospinota* based on the analyses of MAGs and genomes of cultivated representatives. Our aim was to comprehensively investigate the metabolic capabilities and flexibility of this phylum, with the emerging questions of whether all *Nitrospinota* possess the ability to oxidize nitrite and which other metabolic traits might be employed within this phylum. Overall, our (meta)genome-based approach revealed that members of the phylum *Nitrospinota* are much more metabolically versatile than previously anticipated, and not all appear to be nitrite-oxidizers.

## Materials and methods

### Dataset compilation

We used 315 *Nitrospinota* genomes, 43 of which were derived from the OceanDNA dataset [15], 36 from the published GEM catalog [17], 2 from the Caspian Sea, and 1 from the enrichment culture MSP, with the latter three published by Park *et al.* [11]. The remaining genomes were downloaded from NCBI and IMG (Table S1). Completeness and redundancy of the genomes were assessed with CheckM (v1.0.11; Table S1) [30]. Nonredundant genomes were selected using dRep with an average nucleotide identity (ANI) cutoff ≥99%, the “average” clustering algorithm and “ANImf” for secondary clustering (v2.4.2; Table S3) [31]. Dereplicated medium-quality (completeness >75%, redundancy <10%) and high-quality (completeness >90%, redundancy <5%) genomes were retained for further analyses. For all medium- and high-quality genomes, shortened names were used throughout the text and figures. A list of their full names is provided in Table S2.

### Classification and phylogenomic tree reconstruction

*Nitrospinota* genomes were classified using the Genome Taxonomy Database Toolkit (v1.6.0) classification workflow (classify_wf) with the r207 reference database [32]. Please note that the classification of two class UBA9942 MAGs has changed and that new genomes and taxonomic groups have been added with the new release of the GTDB r214 reference database. ANI and average amino acid identity (AAI) values of high-quality, dereplicated genomes were calculated [33] and visualized in R (v3.6.2) [34] using the geom_tile function of the ggplot2 (v3.3.5) package [35].

The retained medium- and high-quality genomes (Table S2) were used to construct phylogenomic trees of the *Nitrospinota* based on the concatenated alignments of 92 core genes, of which all genomes had ≥44 genes, using the UBCG pipeline (v3.0) [36]. The genomes of the following four *Nitrospira* species served as outgroup: *N. moscoviensis* NSP M-1 (GCF_001273775.1), *N. inopinata* ENR4 (GCF_001458695.1), *N. japonica* NJ11 (GCF_900169565.1), and *N. defluvii* (GCF_000196815.1). IQ-Tree (v1.6.12) ModelFinder identified GTR + F + I + G4 as the best fitting model for the high-quality genomes and SYM + I + G4 for the medium-quality genome dataset [37]. The final trees were constructed using IQ-Tree (1.6.12) with 1000 ultra-fast bootstrap replicates [38]. Trees were visualized and annotated using Interactive Tree of Life (iTol, v6) [39].

### Annotation of *Nitrospinota* genomes

DRAM [40] was used for gene calling with prodigal [41] and annotation against the KOfam [42], UniRef90 [43], Pfam [44], and dbCAN [45] databases. Key genes were manually curated using blastp (2.13.0+) [46] searches of representative proteins (e-value <0.00001, bitscore >30, percent identity >30%). Protein complex and pathway completeness were calculated based on the presence of the minimum number of genes required. Putative [NiFe] hydrogenases were identified in the annotation based on Pfam accession PF00374 and classified using HydDB [47]. The type of succinate:quinone oxidoreductase was identified by the presence of the conserved cysteine motif CX_31_CCGX_34_CX_2_C, followed by a CX_39_CCGX_34_CX_2_C motif (type E) [48] and based on InterProScan 5 search results (IPR000701, type B) [49]. Marker proteins for iron metabolism were searched with FeGenie [50]. Mercury methylation proteins were searched using the HgcA HMM model of the Mercury Methylator Database [51], while retaining only sequences containing the N(V/I)WCA(A/G) motif [52]. The sequence motifs C(M/I)ECGA and the tandem CXXCXXC motif were then used to identify HgcB encoding genes on the same contigs [52, 53]. Signal sequences for the twin-arginine translocation (Tat) pathway (IPR006311) in NxrA sequences were identified using the search function of SignalP 6.0 [54]. CRISPR arrays and Cas proteins were identified using the online CRISPRCasFinder (https://crisprcas.i2bc.paris-saclay.fr/) with default settings [55]. Annotations for iron metabolism, stress resistance and osmoprotection, and CRISPR arrays and Cas proteins are summarized in the Supplementary Text, Figs. S9 and S10, and Tables S6 and S7.

### Phylogenetic analysis of protein sequences

The amino acid sequences of key proteins (NxrA/NarG, DsrA, and DsrB) were retrieved from the manually curated DRAM annotations of the medium- and high-quality nonredundant genomes. For NxrA/NarG, 33 sequences with a minimum length of 850 amino acids were aligned to the reference dataset described by Poghosyan *et al.* [56] using the software package ARB v5.5 [57]. The final alignment included 413 sequences and was trimmed by compressing vertical gaps, resulting in 1820 alignment positions of which 1746 were distinct patterns. For DsrAB, a subsampled dataset of sequences by Pelikan *et al.* [58] was used to construct the DsrAB tree, with additional sequences from *Candidatus* Sulfobium mesophilum [59] and *Candidatus* Nitrobium versatile [60]. DsrAB sequences were aligned using muscle (v3.8.31) [61], and the alignments were trimmed using trimAl (v1.4.rev22), removing all positions with gaps in more than 5% of the sequences (−gt 0.95) [62]. Maximum likelihood trees were constructed using IQ-Tree (1.6.12) or the online tool W-IQ-Tree (for NxrA) including ModelFinder with 1000 ultra-fast bootstrap replicates [37, 38, 63]. ModelFinder determined LG + I + G4 (NxrA) and LG + F + G4 (DsrAB) to be the best-fitting models.

*Nitrospinota* CydA sequences identified by DRAM were added to a multiple sequence alignment (MSA2) of CydA sequences encoding quinol-oxidizing *bd*-type O_2_ reductases by Murali *et al.* [64] and aligned with muscle [61]. Alignment trimming and phylogenetic tree calculation were performed as described above, using the VT + F + G4 model. All trees were visualized and annotated using iTol (v6) [39].

### 16S rRNA gene phylogeny

All 16S rRNA gene sequences detected by DRAM were extracted from the *Nitrospinota* genomes. Additional *Nitrospinota* 16S rRNA gene sequences were retrieved using hmmsearch (http://hmmer.org/) with the barrnap 16S rRNA gene HMM model (v.0.9) (available from: https://github.com/tseemann/barrnap). Reference sequences were downloaded from the SILVA SSU Ref NR database r138.1 [65] with the following criteria: taxonomy: *Nitrospinota*; sequence length: >1399 nucleotides; sequence quality score: >90; pintail score: >90. Additional relevant reference sequences were selected based on previously published *Nitrospinota* 16S rRNA gene trees [5, 6, 11, 12] and identified by blasting the *Nitrospinota* 16S rRNA gene sequences from the medium- and high-quality MAGs against the NCBI nt database. The reference sequences were combined and filtered by length (>1400 bp, <1600 bp) before clustering with the cluster_fast command of usearch (v11.0.667) [66] using an identity threshold of 0.95. Two additional sequences were retrieved from the MiDAS 4.8.1 database [67] and added after clustering. The reference sequences were combined with the MAG-derived *Nitrospinota* 16S rRNA gene sequences. Near-full-length sequences (>1400 bp) were aligned using the SINA aligner [68]. The alignment was trimmed using trimAl (v1.4.rev22), removing all positions with gaps in more than 5% of the sequences (−gt 0.95) [62]. Trees were constructed using IQ-Tree (v1.6.12) including ModelFinder with 1000 ultra-fast bootstrap replicates [37, 38] with the SYM + I + G4 model, and visualized and annotated using iTol (v6) [39]. The 16S rRNA gene sequences of *N. defluvii* (FP929003), *N. moscoviensis* (X82558), and *N. marina* Nb-295 (X82559) were used as outgroup.

Minimum and average sequence similarities of the 16S rRNA sequences in the alignment were calculated using SIAS (Sequences Identities And Similarities, http://imed.med.ucm.es/Tools/sias.html) using default settings.

## Results and discussion

**Figure 1:**
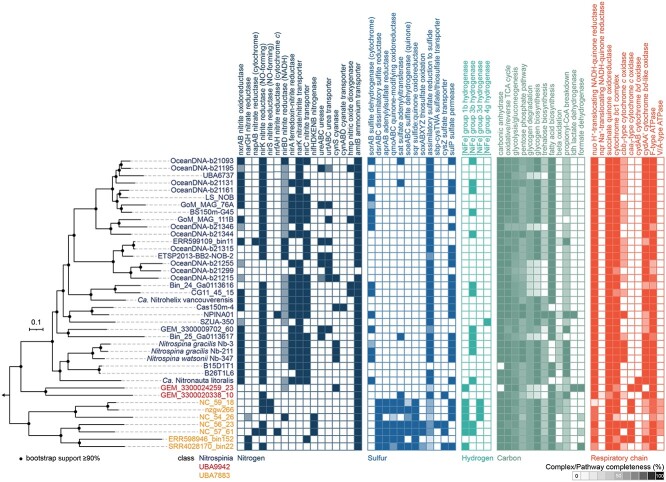
Heatmap showing presence of key genes involved in high-quality *Nitrospinota* genomes in energy metabolism, and nitrogen and sulfur assimilation; on the left, a phylogenomic tree of dereplicated high-quality *Nitrospinota* genomes (>90% completeness, <5% redundancy) based on concatenated alignments of 92 core protein sequences is shown; the maximum likelihood tree was calculated using IQ-tree with the GTR + F + I + G4 model selected by the IQ-tree ModelFinder; filled circles represent bootstrap support ≥90% of 1000 ultrafast bootstrap replicates; presence and completeness of marker genes and pathways are shown in the heatmap; the shade represents the completeness of the pathway based on the number of subunits identified; some MAG names were shortened, see Table S1 for full genome names and accession numbers; for more detailed information on the genome analysis, see Table S4 and Fig. S5.

All cultured members of the *Nitrospinota* phylum are described as aerobic chemolithoautotrophic bacteria that conserve energy by nitrite oxidation to nitrate and fix CO_2_ as their sole carbon source [9–12, 25]. Here, we show potential metabolic versatility in the phylum, with the capacity for not only nitrite but also hydrogen and formate oxidation, as well as the oxidation of reduced sulfur compounds (Fig. 1, Table S4). The dominant carbon source is CO_2_, based on the high degree of conservation of the reductive TCA (rTCA) cycle in all analyzed dereplicated medium- and high-quality *Nitrospinota* genomes (Table S5).

As with other (meta)genomics-based studies, our results must be interpreted keeping in mind that it is unknown whether missing genes are due to incomplete genomes caused by binning, assembly, or sequencing or whether these organisms truly lack these functions.

### General genomic features and phylogeny

A total of 315 genomes were dereplicated at 99% ANI values, resulting in 98 nonredundant medium- to high-quality *Nitrospinota* genomes. Based on their estimated completeness and redundancy values (>90% and <5%, respectively) [30], 40 high-quality genomes with an average length of 2.75 ± 0.56 Mbp were chosen for detailed analyses (Fig. 1).

**Figure 2:**
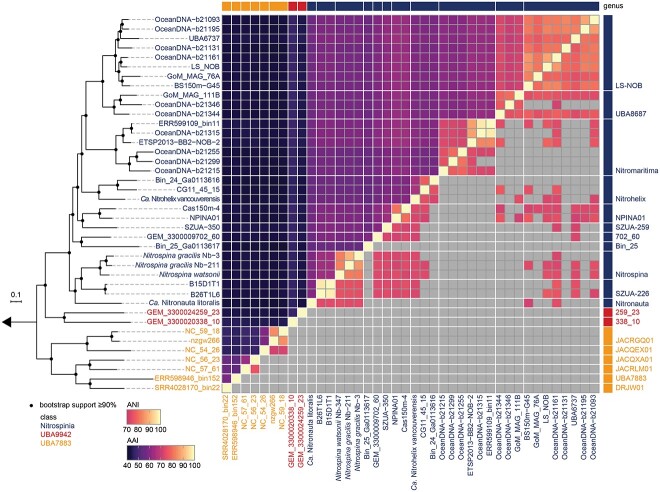
Average amino acid and nucleotide identities of *Nitrospinota* genomes; on the left, the same phylogenetic tree as in Fig. 1 is shown; the upper-left part of the heatmap shows average AAI values and the lower-right part ANI values ≥70%; ANI values <70% are replaced by gray squares; the taxonomic information (class, genus) is based on GTDB-Tk classifications and AAI values.

The phylum *Nitrospinota* is very diverse as shown by the low-average AAIs and the classification by the Genome Taxonomy Database (GTDB; Fig. 2). Our analyses confirmed the six different classes of *Nitrospinota* as found in the GTDB classification: *Nitrospinia*, UBA9942, UBA7883, 2-12-FULL-45-22, JACRGO01, and JADGAW01, which can be further divided into at least 11 families and 33 genera (Fig. 2, Table S1). Previous analyses indicated that the phylum *Nitrospinota* might not be monophyletic [69]. According to the GTDB taxonomy version r207 based on the concatenated alignment of 53 single copy genes, *Nitrospinota* genomes belonging to the class 2-12-FULL-45-22 cluster with genomes from the phyla Tectomicrobia, JACPUC01, JACPSX01, and UBA8248. Since the class 2-12-FULL-45-22 clustered with the Tectomicrobia, the phylum *Nitrospinota* was split into two phyla according to the GTDB classification, with the class 2-12-FULL-45-22 assigned to the Nitrospinota_B (Fig. S1A). However, in our phylogenomic analysis based on the concatenated alignment of 92 core genes, the MAG RIFCSPLOWO2_45_22 (class 2-12-FULL-45-22) falls within the class UBA9942 and was thus included in the analyses as part of the phylum *Nitrospinota* (Fig. S1B). In addition, the median sequence identity of the 16S rRNA genes of the analyzed *Nitrospinota* sequences (Fig. S2) is 85.10%, which is above the median sequence identity of 83.68% and the taxonomic threshold of 75% identity for phyla determined by Yarza *et al.* [70], supporting the placement of class 2-12-FULL-45-22 within the phylum *Nitrospinota*. We note that, although GTDB provides a standardized and reproducible taxonomic classification, future efforts are needed to reevaluate the taxonomy of this highly diverse phylum.

The class *Nitrospinia* contains the cultured nitrite-oxidizing representatives *Nitrospina gracilis* Nb-3*, Nitrospina gracilis* Nb-211, *Nitrospina watsonii* Nb-347, *Ca.* Nitronauta litoralis, *Ca.* Nitrohelix vancouverensis, and the enrichment culture MSP [9–12]. In contrast, the other classes (UBA9942, UBA7883, 2-12-FULL-45-22, JACRGO01, and JADGAW01) lack cultured representatives.

### Distribution in marine and groundwater habitats

Most of the analyzed high-quality *Nitrospinota* genomes originated from marine habitats or marine cultures (*n* = 34) and contained genomes derived from sponge metagenomes (*n* = 2), sediments or microbial mats (*n* = 5), brackish water (*n* = 4), and waters with low O_2_ concentrations (*n* = 8). Still, in total, six of the analyzed high-quality *Nitrospinota* genomes were obtained from different groundwater metagenomes from Calistoga and Middletown (California), Green River (Utah), and the Canterbury region in New Zealand [19, 21, 71]. Most MAGs from the classes UBA9942, 2-12-FULL-45-22, and UBA7883 originate from groundwater sites, but 16S rRNA gene phylogeny shows that closely related *Nitrospinota* also do occur in marine habitats, with many sequences obtained from hydrothermal vents (Fig. S2).

Members of the *Nitrospinia* family are most commonly found in marine environments, but also *Nitrospinia* MAGs were recovered from groundwater samples [19, 21, 71]. Intriguingly, in a limestone aquifer, the dominant OTU that constituted 21% of the microbial community [72] had 99% sequence similarity to a 16S rRNA gene sequence from an uncultured bacterium found in lake sediment (AB661566), which clusters with other *Nitrospinia* sequences in the 16S rRNA gene tree (Fig. S2). This indicates that this limestone aquifer was dominated by *Nitrospinia* species that remained unidentified, as the authors did not perform phylogenetic tree reconstruction. However, a search of the *Nitrospinota* sequences deposited in the SILVA NR r138.1 database did not reveal a widespread occurrence of *Nitrospinia* in groundwater samples.

### Carbon metabolism

All core features for autotrophic growth using the rTCA cycle for CO_2_ fixation are conserved in the phylum *Nitrospinota*. The genomes contain all required genes for the reductive and oxidative tricarboxylic acid (TCA) cycles, glycolysis and gluconeogenesis, and the pentose phosphate pathway (Fig. 1, Table S5). Notably, in contrast to D’Angelo and coworkers [29] who report the lack of 2-oxoacid oxidoreductases in later branching clades in the *Nitrospinota* and *Nitrospirota*, we confirm the presence of this key rTCA enzyme family in all *Nitrospinota* classes including *Nitrospinia* as described earlier (Fig. S3, Table S5) [25]. In addition, all but two of the high-quality *Nitrospinota* genomes encode a carbonic anhydrase, which converts carbonic acid (HCO_3_^−^) to CO_2_ for autotrophic carbon fixation (Fig. 1). Consistent with the observation of *N. gracilis* containing glycogen deposits [9], many *Nitrospinota* genomes encode genes for glycogen biosynthesis. Most of those organisms are also capable of trehalose synthesis from glycogen (Figs.1 and Fig. S4).

Several MAGs belonging to the classes *Nitrospinia* and UBA9942 contain genes for propionyl-CoA breakdown to succinate (Fig. 1). In addition, an NAD^+^-dependent lactate dehydrogenase is encoded in three MAGs (nPCRbin9 [class JADGAW01], NPINA01 [class *Nitrospinia*], GEM_3300024259_23 [class UBA9942]; Fig. S4), enabling them to reversibly oxidize lactate to pyruvate, which might either enter the central carbon metabolism or might play a role in H_2_O_2_ detoxification as shown for other nitrifiers [73, 74]. Lastly, two of the high-quality *Nitrospinota* MAGs encode an NAD(P)^+^-dependent formate dehydrogenase (GEM_3300024259_23 [class UBA9942] and SRR4028170_bin22 [class UBA7883]), enabling them to oxidize formate to CO_2_, which could be coupled to the reduction of the electron acceptors O_2_ or nitrate as previously reported for *N. moscoviensis* [24].

### Terminal oxidases

A recent study showed that there are three large gene families within the cytochrome *bd-*type O_2_ reductase superfamily that differ in their subunit composition and presence of quinol and heme-binding sizes [64]. Based on the CydA phylogeny (Fig. S5), the putative cytochrome *c*-oxidizing OR-N type CydAA’ O_2_ reductase is conserved in most *Nitrospinota*. Contrastingly, only three of the *Nitrospinota* MAGs encode a canonical qOR-type quinol-oxidizing cytochrome *bd* O_2_ reductase: GEM_3300024259_23 (class UBA9942, qOR1-type), HKST-UBA01, and *Ca.* Nitronauta litoralis (class *Nitrospinia*, qOR2-type), with the latter two also containing OR-N-type CydA sequences. The MAG OceanDNA-b21356 (class UBA9942) encodes a CydA that clusters at the base of the OR-C and OR-N clades. All other CydA sequences represent OR-N-type enzymes that belong to the OR-N1 and OR-N2 clades. Although *Nitrospinia* CydA sequences form separate clusters within these clades, there is no class-specific clustering of the other *Nitrospinota* sequences (Fig. S5). Cytochrome *bd*-type O_2_ reductases belonging to the OR-N family likely use cytochrome *c* as the electron donor instead of quinol and it was hypothesized that OR-N1 and OR-N2 CydA are associated and form a CydAA’ complex [25, 64, 75]. Although experimental confirmation of the exact subunit composition and proposed function as potentially proton-pumping O_2_ reductases (terminal oxidases) are still lacking, the complex likely exhibits a high O_2_ affinity, considering its similarity to canonical high-affinity cytochrome *bd* oxidases and the high abundance of *Nitrospinia* in O_2_-depleted systems such as OMZs [1, 4]. In addition to being used as terminal oxidases for aerobic respiration with high O_2_ affinity [76], canonical cytochrome *bd* O_2_ reductases (qOR) can also play a role in O_2_ and nitric oxide detoxification [77–79], particularly for the protection of O_2_-sensitive nitrogenases [80, 81] and ferredoxin-dependent components of the rTCA cycle [82]. It remains to be determined whether this is also the case for the yet uncharacterized OR-N enzyme family.

Several *Nitrospinota* MAGs also encode heme-copper oxidases of the high-affinity *cbb_3_* or the low-affinity *caa_3_-*type (Fig. 1 and Fig. S4). *N. gracilis* encodes a *cbb_3_*-type heme copper cytochrome *c* oxidase, whose three subunits are fused into one gene [25]. Notably, *cbb_3_*-type terminal oxidases were only found in a few of the class *Nitrospinia* genomes analyzed here: *N. gracilis* Nb-3, *N. gracilis* Nb-211, *Nitrospina watsonii* Nb-347, *Nitrospinia* enrichment MSP, and in the MAG NPINA01 recovered from an artificial seawater bioreactor. In addition, unfused *cbb_3_*-type terminal oxidases were found in four class UBA7883 MAGs (NC_59_16, ERR598946_bin152, nNGHbin12, NC_56_23; Fig. 1 and Fig. S4). Low-affinity heme-copper oxidases of the *caa_3_*-type were identified in most UBA7883 MAGs and in some MAGs belonging to the classes UBA9942, JADGAW01, JACRO01, and 2-12-FULL-45-22. Subunit I of cytochrome *o* ubiquinol oxidase was found in two class *Nitrospinia* MAGs (OceanDNA-b21299 and SZUA-350), both on the ends of a contig. Such presence of several distinct terminal oxidases may be advantageous under fluctuating environmental conditions, allowing for a broader habitat range [83, 84].

### *Nitrospinia* differ from other classes of *Nitrospinota*

As mentioned above, members of the phylum share some common features like the rTCA cycle for carbon fixation and the cytochrome *bd*-type O_2_ reductases. However, although members of the class *Nitrospinia* were described as key nitrite oxidizers in various marine systems [6] and we identified all core features for autotrophic nitrite oxidation that were discussed in detail elsewhere in the analyzed genomes of cultured *Nitrospinia* species [11, 12, 25], organisms belonging to the other *Nitrospinota* classes differ significantly in gene content from the nitrite-oxidizing *Nitrospinia*, as we will discuss in detail below. These classes (UBA9942, UBA7883, 2-12-FULL-45-22, JADGAW01, JACRGO01) lack cultured representatives, were mainly recovered from subsurface habitats, and resemble the predicted metabolic makeup of ancestral *Nitrospinota* [29].

### Respiratory chain

All analyzed *Nitrospinota* genomes encode genes for the five complexes of the respiratory chain (Fig. 1 and Fig. S4, Suppl. Text). Due to the high redox potential of the nitrite/nitrate couple (E^0′^ = +0.42 V), nitrite-oxidizing *Nitrospinia* transfer the electrons derived from nitrite oxidation at the NXR via cytochrome *c* directly to a terminal oxidase [25]. The other respiratory chain complexes are used to generate reducing equivalents via reverse electron transfer and, especially in the other *Nitrospinota* classes to couple the oxidation of other electron donors such as glycogen, sulfide, or H_2_ to the reduction of O_2_ or another suitable electron acceptor.

The genomes belonging to the class *Nitrospinia* do not only encode a canonical NADH:quinone oxidoreductase (NUO-1, Complex I) but also a complete second set of *nuo* genes elsewhere in the genome (Fig. S6), as was previously described for the cultured representative *N. gracilis* [25]. It was speculated that the canonical NUO-1 could transport electrons from NADH to quinone, while the NUO-2 might be involved in reverse electron transport from quinol to ferredoxin. In contrast, most *Nitrospinota* belonging to other classes lack this second set of *nuo* genes. For autotrophic growth, these non-*Nitrospinia* members are therefore likely restricted to utilizing electron donors with a much lower reduction potential than nitrite, which facilitate the reduction of the low-potential ferredoxins needed in the rTCA cycle [85] or contain yet unknown ferredoxin-reducing complexes.

Although all *Nitrospinota* harbor an F_1_F_o_-type ATP synthase (ATPase, Complex V), some MAGs (classes UBA9942, UBA7883, JACRGO01), most of which were recovered from subsurface metagenomes, encode a second ATPase of the bacterial V-type (also named V/A-ATPase; Fig. 1 and Fig. S4) that is closely related to the archaeal A-type ATPase and probably was transferred from archaea to bacteria through horizontal gene transfer [86]. These ATPases are composed of subunit A and B, forming the soluble V_1_ domain, the subunits I (also named subunit a) and K (also named L or c), forming the ion-translocating V_o_ domain, and the subunits C (also named d), D, E, F, and G, which form the connecting stalk region. Similar to *Euryarchaeota* genomes, the genes occur in the order *atpIKECFABD* [86, 87]. Subunit G could not be identified in the genomes, which might be due to low sequence similarities to known sequences. There are multiple subtypes of prokaryotic V/A ATPases, and variation in subunit composition may play a role in adaptation to environmental conditions such as hydrostatic pressure or pH [88]. However, whether the second ATPase encoded in the subsurface *Nitrospinota* MAGs functions in ATP hydrolysis or synthesis remains unclear.

### Dissimilatory nitrogen metabolism

The key enzyme for nitrite oxidation, NXR, is used as a functional and phylogenetic marker for NOB [89]. However, several phylogenetically distinct NXR isoenzymes are known and often show a high sequence similarity to strict nitrate reductases (NARs), making it difficult to distinguish between NXR and NAR activity based on sequence similarity and phylogeny [89]. Based on NxrA/NarG phylogeny (Fig. S7), *Nitrospinota* bacteria possess different types of nitrite oxidase/NAR-like enzymes. All NxrA sequences extracted from *Nitrospinia* MAGs except for the medium-quality MAG ERR599109_bin11 cluster with *Nitrospina* NxrA sequences that oxidize nitrite to nitrate. Still, not all analyzed *Nitrospinia* genomes contained *nxrA* and *nxrB* genes, but as these genes often occur in multiple highly similar copies in the genomes of nitrite oxidizers, they are difficult to bin based on sequence coverage. Consistent with previous analyses, most complete sequences of NXR subunit alpha (NxrA) contained a twin arginine motive for the translocation into the periplasm. The NXR of *Nitrospinia* is thus located in the periplasm, as is the case for nitrifiers belonging to the class *Nitrospiria* [24, 75, 90, 91].

All other *Nitrospinota* NxrA/NarG sequences either belong to clusters that contain both NxrA and NarG sequences [92, 93], preventing their functional classification as NXR or NAR without physiological evidence, or they are affiliated with known NarG sequences and thus are probably strict NARs. Notably, the MAG OceanDNA-b21356 clusters outside the *Nitrospinia* NxrA but is still included in the *Nitrospira*/*Nitrospina*/anaerobic ammonium oxidation (anammox) bacteria NxrA branch of the tree. Since this medium-quality MAG is the only representative of class UBA9942 possessing this enzyme type, it is unclear whether members of this class might be able to oxidize nitrite or whether this enzyme was wrongly assigned to this MAG due to misbinning. The phylogenetic placement of these enzymes thus indicates that the *Nitrospinia* can oxidize nitrite to nitrate, whereas the NXR/NAR-like proteins of the bacteria belonging to the other classes likely catalyze nitrate reduction rather than nitrite oxidation. Taken together, the NXR/NAR phylogeny, as well as the observed lack of assimilatory nitrite reductases and the presence of other metabolic traits such as hydrogen, sulfide, or thiosulfate oxidation (see below), suggest an ecophysiological role for these non-*Nitrospinia Nitrospinota* outside nitrification.

Most *Nitrospinota* genomes contain a conserved copper-containing nitrite reductase (NirK), and some class UBA7883 MAGs also encode a heme-containing nitrite reductase (NirS), both of which may allow them to further convert nitrite to nitric oxide (Fig. S4). The *norBC* and *nosZ* genes for the final steps of denitrification are absent from all genomes we analyzed. Thus, nitrate may serve as terminal electron acceptor during growth on substrates other than nitrite but will not be reduced further than nitrite or possibly nitric oxide.

A previous study reported that the SAG of *Candidatus* Nitromaritima RS recovered from the Red Sea encodes a periplasmic nitrate reductase (NAP) [5]. According to our analyses, only four of the analyzed *Nitrospinota* MAGs contain *napAB* genes and, additionally, NC_41_11 (class UBA9942) encodes NapA only. Thus, nitrate reduction catalyzed by the NAP complex does not seem to be a widespread feature in the *Nitrospinota* phylum. Next to an NAP, the MAG NC_57_61 (class UBA7883) encodes a cytochrome *c* nitrite reductase (NrfAH), potentially enabling it to perform dissimilatory nitrate reduction to ammonium (DNRA). Five other MAGs also encode Nrf-type nitrite reductases but lack *nap* genes (NPINA01, B2T1L10, CG11_56_8, and MSP [class *Nitrospinia*]; NC_59_45 [class UBA7883]; Fig. S4). Even if only identified in the enrichment culture MSP MAG, those *Nitrospinia* genomes are assumed to all encode NxrAB. This would enable them to convert nitrate to nitrite, which subsequently could be reduced to ammonium by NrfAH. Thus, although not widespread, few *Nitrospinota* may be able to perform DNRA under anoxic conditions.

### Assimilatory nitrogen metabolism

Most *Nitrospinota* bacteria can take up external ammonium via an AmtB-type transporter. Additionally, some members of this phylum can produce ammonium for assimilation via different anabolic reactions, including nitrite reduction, or cyanate and urea breakdown. A characteristic feature distinguishing the *Nitrospinia* from the other lineages of the phylum *Nitrospinota* is their capability for assimilatory nitrite reduction to ammonium using NirA, which is encoded in most *Nitrospinia* genomes. Although most MAGs also contain the NirD subunit of the NirBD-type nitrite reductase, the presence of the catalytic subunit NirB is rare (Fig. 1 and Fig. S4). Similar to *Nitrospinia*, assimilatory nitrite reductases have been identified for most nitrite oxidizers with the exception of one *Nitrotoga* and several *Chloroflexota* species, and the complete ammonia oxidizers (comammox) within the genus *Nitrospira* [92, 94–97].

In addition to nitrite reduction, *Nitrospinia* representatives possess the genomic potential for cyanate breakdown to ammonium catalyzed by cyanase (CynS; Fig. 1 and Fig. S4). In the other *Nitrospinota* classes, only a single-class UBA9942 MAG (GEM_3300024259_23) encodes CynS. ABC transporters putatively involved in cyanate transport were found next to the *cynS* gene the MAGs OceanDNA-b21351 and Cas150m-4. Contrastingly, the MAGs SI034_bin134, OceanDNA-b21351, OceanDNA-b21342, OceanDNA-b21215, OceanDNA-b21154, and ALOHA_A20_37 (class *Nitrospinia*) encode a NirC-type nitrite transporter adjacent to the *cynS* gene, which also might be involved in cyanate uptake [98]. Additionally, many *Nitrospinia* MAGs possess ureases (UreABC) and a high-affinity urea ABC transport system (UrtABCDE), which are absent in the other *Nitrospinota* classes (Fig. 1 and Fig. S4). Numerous studies have shown that many NOB encode these proteins [6, 99] and use them to produce ammonium from organic N compounds for assimilation [100]. The produced ammonium might also be used by NOB in a reciprocal feeding mechanism with ammonia oxidizers lacking these enzymes [24, 98]. Especially in OMZs, which often contain ammonium concentrations below 0.1 μM but high *Nitrospinia* abundances, such a potential to utilize organic N compounds for assimilation and potential reciprocal feeding interactions may confer a selective advantage [1, 101, 102].

As nitrogen availability is a growth-controlling factor in most habitats, the uptake and utilization of alternative nitrogen sources besides ammonium are a vital niche-defining factor. Although proteins for the import and breakdown of nitrite and organic nitrogen compounds are lacking in non-*Nitrospinia*, some class UBA9942, UBA7883, and JADGAW01 members possess the enzymatic repertoire for nitrogen fixation, which is lacking in *Nitrospinia* [29]. The *nifHDK* and *nifENB* genes are coding for the structural and biosynthetic components of the nitrogenase required for N_2_ fixation [103]. Three class UBA9942 (NC_39_25, NC_39_30, SZUA-224) and four class UBA7883 MAGs (NC_54_26, NC_63_8, NC_56_23, NC_57_61) encode all of these in addition to regulatory *nif* genes (Fig. 1 and Fig. S4). The class JADGAW01 MAG nPCRbin9 encodes five of the six required genes, along with regulatory *nif* genes, but lacks *nifN*. These nitrogenase-containing *Nitrospinota* MAGs were obtained from a hydrothermal vent (SZUA-224), a river sediment (nPCRbin9), and subsurface metagenomes [19, 20]. Diazotrophs in subsurface environments and marine (deep-sea) sediments have been proposed to be phylogenetically diverse and crucial for supporting growth in these ecosystems [104, 105]. In a recent study employing both ^15^N-DNA stable isotope probing and *nifH* amplicon sequencing on deep marine sediments, *Nitrospinota* were found to be among the ^15^N-incorporating organisms, but no *nifH* sequences were associated with the phylum [105]. However, it is conceivable that *Nitrospinota nifH* sequences were not detected due to primer bias, lack of reference sequences, or insufficient sequencing depth [105]. Thus, diazotrophic *Nitrospinota* might provide fixed nitrogen for biomass production in ammonium-limited environments.

### Sulfur metabolism

The potential for sulfur cycling was previously observed in the marine NOB *N. mobilis* [8] and for non-nitrite-oxidizing members of the phylum *Nitrospirota* [59, 60]. Some of the class *Nitrospinia* genomes encode sulfite:ferricytochrome *c* oxidoreductases (SorAB; Fig. 1 and Fig. S4) that may enable them to use sulfite as an alternative energy source, as was previously hypothesized for *N. gracilis* [25]. However, physiological evidence of sulfite oxidation by the nitrite-oxidizing *Nitrospinia* is still lacking.

**Figure 3:**
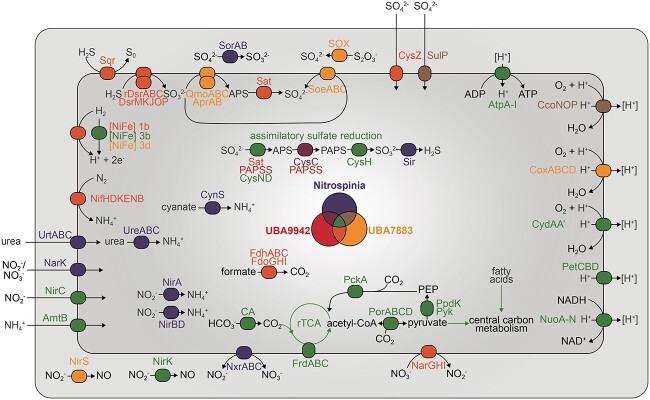
Cell cartoon showing the energy metabolism and nitrogen and sulfur acquisition of the classes *Nitrospinia*, UBA9942, and UBA7883; proteins, complexes, and pathways are shown if one or more genes are present in at least 20% of the MAGs belonging to the respective class; for more detailed information on the annotation, see Table S4 and Fig. S5; AmtB, ammonium transporter; AprAB, adenylylsulfate reductase; AtpA-I, F-type H + -transporting ATPase; CA, carbonic anhydrase; CcoNOP, *cbb_3_*-type cytochrome *c* oxidase; CoxABCD, *caa_3_*-type cytochrome *c* oxidase; CydAA’, cydAA’ cytochrome *bd*-like oxidase; CynS, cyanate lyase; CysC, adenylylsulfate kinase; CysH, phosphoadenosine phosphosulfate reductase; CysND, sulfate adenylyltransferase; CysZ, sulfate transporter; FdoGHI/FdhABC, NAD^+^/NADP^+^-dependent formate dehydrogenase; NarGHI, nitrate reductase; NarK, nitrate/nitrite transporter; NifHDKENB, nitrogenase; [NiFe] 1b, [NiFe] hydrogenase group 1b; [NiFe] 3b, [NiFe] hydrogenase group 3b; [NiFe] 3d, [NiFe] hydrogenase group 3d; NirA, assimilatory ferredoxin-nitrite reductase; NirBD, assimilatory NADH-dependent nitrite reductase; NirC, nitrite transporter; NirK, NO-forming nitrite reductase; NirS, NO-forming nitrite reductase; NuoA-N, NADH-quinone dehydrogenase; NxrABC, nitrite oxidoreductase; PAPSS, 3′-phosphoadenosine 5′-phosphosulfate synthase; PckA, phosphoenolpyruvate carboxykinase; PEP, phosphoenolpyruvate; PetCBD, cytochrome *bc_1_* complex; PorABCD, pyruvate:ferredoxin oxidoreductase; PpdK, pyruvate, orthophosphate dikinase; Pyk, pyruvate kinase; QmoABC, quinone-modifying oxidoreductase; rDsrAB, reverse dissimilatory sulfite reductase; Sat, sulfate adenylyltransferase; SdhCAB, succinate dehydrogenase; Sir, sulfite reductase; SoeABC, sulfite dehydrogenase; SorAB, sulfite dehydrogenase; SOX, Sox enzyme system for thiosulfate oxidation; Sqr, sulfide:quinone oxidoreductase; SulP, sulfate permease; UreABC, urease; UrtABC, urea transport system.

**Figure 4:**
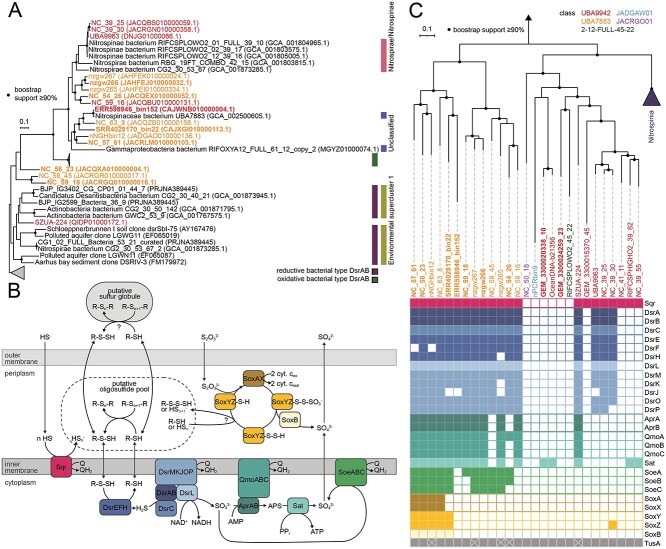
Sulfur metabolism in *Nitrospinota*; (A); phylogeny of the *Nitrospinota* DsrAB enzymes; the unrooted phylogenetic tree was calculated using a subsampled dataset of DsrAB sequences compiled by Pelikan *et al;* [56]; the maximum likelihood tree was calculated using IQ-tree using the LG + F + G4 model; black circles represent bootstrap support ≥90% of 1000 ultrafast bootstrap replicates; all other sequences included in the tree were collapsed for visualization; the complete DsrAB tree can be found in Fig. S6; (B) overview of putative pathways of sulfur metabolism in the *Nitrospinota* classes UBA9942 and UBA7883 adapted from literature [117, 120, 125, 126, 140]; whether sulfur globule formation occurs in the periplasm or outside of the cell is undetermined; transporters were omitted for simplicity; their distribution is shown in Fig. 1 and Fig. S4; (C); phylogenomic tree of dereplicated medium- and high-quality *Nitrospinota* genomes based on concatenated alignments of 92 core protein sequences; the maximum likelihood tree was calculated using IQ-tree with the GTR + F + I + G4 model; black circles represent bootstrap support ≥90% of 1000 ultrafast bootstrap replicates; presence of genes involved in sulfur metabolism is shown in the presence/absence matrix using the same color code as in (B); *TusA* genes that are encoded next to the *dsrE* gene are marked with an X; names from *Nitrospinota* MAGs with >90% completeness and <5% redundancy are shown in bold; some names were shortened; see Table S1 for full genome names and accession numbers; AprAB, adenylylsulfate reductase; QmoABC, quinone-modifying oxidoreductase; DSR, dissimilatory sulfite reductase enzyme complex; Sat, sulfate adenylyltransferase; Sir, sulfite reductase; SoeABC, sulfite dehydrogenase; SorAB, sulfite dehydrogenase; SOX, Sox enzyme system for thiosulfate oxidation; Sqr, sulfide:quinone oxidoreductase.

In contrast, the other *Nitrospinota* classes most likely use sulfide and thiosulfate oxidation for energy conservation (Figs 1, 3, and 4), as suggested by D’Angelo and coworkers [29], and indicated by the presence of dissimilatory sulfite reductase (*dsr*) genes in class UBA9942 MAGs recovered from a subsurface metagenome [20, 27]. The DSR system can be used for dissimilatory sulfite reduction or work in the reverse direction catalyzing the oxidation of reduced sulfur compounds, relying on largely the same enzyme complexes operating in opposite directions [106, 107]. If the DSR complex works in the oxidative direction, the first part of the pathway is the two-step oxidation of sulfide to sulfite involving the DsrC protein, the DsrAB complex, and the membrane-bound DsrMKJOP complex [108–110]. Subsequently, sulfite produced by the DSR complex is oxidized via adenosine 5′-phosphosulfate (APS) to sulfate by the APS reductase (AprAB) and the ATP-producing sulfate adenylyltransferase (Sat). The electrons from sulfite are transferred to the quinone pool via the membrane complex QmoABC [111–113]. These genes are all present in MAGs of class UBA7883 and in several MAGs of the class UBA9942 (Figs 1, 3, and 4), although the complete set of *aprAB* and *qmoABC* genes was only found in one of the UBA9942 MAGs (Fig. 4C).

In a phylogenetic tree based on concatenated DsrAB sequences, most *Nitrospinota* proteins cluster to oxidative bacterial type DsrAB (Fig. 4A and Fig. S8). However, phylogenetic analyses alone may not be sufficient to distinguish between oxidative and reductive DsrAB types [106]. Therefore, the presence of the iron–sulfur flavoprotein DsrL or the sulfur transferase DsrEFH is often additionally used to predict the metabolic direction. Although these proteins are generally considered to be indicative of sulfur oxidation, there are some exceptions [114]. The *dsrL* gene has been found to be essential and highly expressed in sulfur oxidizers [115, 116], but the role of DsrL mainly depends on its physiological context. In the phototrophic sulfur oxidizer *Allochromatium vinosum*, it transfers electrons from the reverse-operating DsrAB to NAD^+^ [117]*,* while some members of the recently identified DrsL-2 class act as electron donors during reductive sulfur metabolism in concert with the reductive-type DsrAB [114]. Thus, not all types of DsrL proteins can be used to predict the direction of the sulfur metabolism. Notably, the DsrL sequence identified in the *Nitrospinota* MAG UBA9963 clusters with DsrL-2 sequences of bacteria with a reductive-type DsrAB [114]. The *dsrL* gene is present in the *dsrAB*-containing *Nitrospinota* genomes and is located near or adjacent to the *dsrC* gene in the majority of the MAGs, upstream or downstream of the *dsrAB* genes. In addition, the UBA7883 and UBA9942 MAGs contain one to two copies of the *dsrEFH* genes, whose predictive power as markers for sulfur oxidation, however, is under debate [118]. An additional gene often found in sulfur oxidizers is *tusA*, which encodes a protein participating in sulfur transfer to the DsrEFH complex [119, 120]. Moreover, in addition to sulfur oxidation, TusA is involved in various pathways as a sulfur carrier [121]. In the genomes of the class UBA7883 and class UBA9942 *Nitrospinota*, between one and four copies of *tusA*-like genes were found and several of these genes are encoded adjacent to the *dsrE* gene, a gene arrangement that is common in sulfur oxidizers [121, 122].

In addition to the DSR system, the Sox enzyme machinery (SoxABXYZ) is encoded in several UBA7883 MAGs (Fig. 4C), enabling them to oxidize thiosulfate. Notably, the *soxCD* genes are lacking. Without the sulfane dehydrogenase SoxCD, the sulfane sulfur could be transferred from SoxYZ to sulfur globules and subsequently oxidized via the reverse DSR pathway [123–125]. Still, whether sulfur globule formation occurs in class UBA7883 *Nitrospinota* and whether this happens in the periplasm or extracellularly (Fig. 4B) remain to be elucidated. Moreover, all three subunits of the sulfite:quinone oxidoreductase (SoeABC) were found in most analyzed UBA7883 MAGs (Fig. S4). This complex can catalyze the oxidation of sulfite to sulfate in the cytoplasm and thus presents an alternative to the AprAB-Sat system [126]. Lastly, class UBA7883 and UBA9942 MAGs encode a polysulfide-producing sulfide:quinone oxidoreductase (Sqr), which could be involved in sulfide oxidation (Fig. S4) or the detoxification of sulfide [127].

In marine sediments, sulfate reduction is a key process leading to the production of sulfide, which, in turn, can be consumed by sulfide oxidizers under oxic or nitrate-reducing conditions [128, 129]. A similarly active but sometimes cryptic sulfur cycling also takes place in anoxic or suboxic waters of marine OMZs [130, 131]. Two MAGs of the class UBA7883 (UBA7883 and GEM_3300020333_14, excluded from most analyses because of their similarity to MAG ERR598946_bin152) were recovered from the Eastern Tropical South Pacific OMZ [17, 132], suggesting that *Nitrospinota* may also play a role in sulfur cycling in anoxic waters. Furthermore, the MAG SRR4028170_bin22 (class UBA7883) was recovered from a metagenome from hydrothermal fluid in the South Mid Atlantic Ridge, which is known to be a source of sulfide [133]. Thus, our analyses indicate that members of the *Nitrospinota* might constitute thus far overlooked contributors to oxidative sulfur conversion in a range of different habitats.

For the uptake of extracellular sulfate, many *Nitrospinia* and UBA7883 MAGs encode a SulP-type sulfate permease. Several class UBA9942 and UBA7883 MAGs, as well as the two class JACRGO01 and JADGAW01 MAGs, also contain the putative CysZ-type sulfate transporter. In addition, MAGs NC_56_23 and NC_59_45 (class UBA7883) encode an ABC-type sulfate/thiosulfate transporter (CysAUW-Sbp). Although genes for assimilatory sulfate reduction were found in many *Nitrospinota* genomes, the assimilatory sulfite reductase (Sir) is absent from most MAGs of non-*Nitrospinia* classes, making the pathway incomplete (Fig. 3) and indicating a dependency on sulfide to be present in their environment.

### Hydrogenases

In addition to nitrite oxidation, an alternative strategy for energy conservation in the class *Nitrospinia* could be hydrogen oxidation coupled to NAD(P)^+^ reduction using the putatively O_2_-tolerant [134, 135] group 3b [NiFe] hydrogenase encoded in some of the genomes (Fig. 1 and Fig. S4), as previously identified in several *Nitrospinia* genomes [5, 11, 12, 25]. However, physiological evidence for this activity in *Nitrospinia* members is lacking. Furthermore, two of the MAGs recovered from different sediment metagenomes (SZUA_350 and bin1391) encoded group 4 g [NiFe] hydrogenases not previously observed in *Nitrospinia*. However, their putative role in coupling ferredoxin oxidation to H_2_ formation and H^+^/Na^+^ translocation remains to be confirmed [47].

In addition, our analyses show that some non-*Nitrospinia Nitrospinota* MAGs encode additional types of hydrogenases (1b, 3b, and 3d) not previously identified in the phylum (Fig. 1 and Fig. S4). In the classes UBA7883 and UBA9942, several MAGs contain both a group 3b or group 3d and an O_2_-sensitive group 1b [NiFe] hydrogenase. The MAG GEM_3300015370_45 (class UBA9942) is the only MAG encoding an O_2_-tolerant 3c [NiFe] hydrogenase. The single MAG belonging to the class JACRG01 (NC_50_18) even has four different types of [NiFe] hydrogenases encoded in the genome (groups 1a, 1b, 2b, and 3d). An O_2_-tolerant group 2b [NiFe] hydrogenase was also found in the MAG nNHGbin12 (class UBA7883). Overall, although not found in all genomes, hydrogenases are widespread in the phylum *Nitrospinota* and more diverse than previously assumed (Fig. 1 and Fig. S4), but experimental analyses will be needed to assess the metabolic roles of these enzymes. The possession of multiple hydrogenase types might enable some *Nitrospinota* to remain active in ecosystems with fluctuating environmental conditions [136]. Although the membrane-bound group 1b [NiFe] hydrogenases are O_2_-sensitive and thus might be limited to anaerobic respiration, the putatively O_2_-tolerant group 3b and 3d [NiFe] hydrogenases could play a role in generating NADH from H_2_ oxidation under oxic and anoxic conditions [137, 138], although evidence for O_2_-tolerance under mesophilic conditions is still lacking [134].

## Conclusion

*Nitrospinota* have been recognized for their importance in nitrogen and carbon cycling in the ocean [4, 6]. Our analyses show that members of this phylum are more widespread and metabolically versatile than previously recognized, as they have distinct genetic potentials to use nitrite, sulfide, and hydrogen for energy conservation. Some features, such as the autotrophic growth using the rTCA cycle for CO_2_ fixation and the presence of an OR-N type CydAA’ putative O_2_ reductase, are conserved across the *Nitrospinota* phylum. However, although the class *Nitrospinia* plays a key role in the global nitrogen and carbon cycles, other *Nitrospinota* apparently lack the ability to oxidize nitrite and may instead be involved in sulfur cycling, although only physiological tests can validate our metagenome-based hypotheses. Further distinguishing features between the classes are the types of hydrogenases found and the pathways available for nitrogen and sulfur assimilation. In contrast to *Nitrospinia*, all other *Nitrospinota* classes lack cultured representatives, but metagenomic analyses can guide cultivation strategies based on metabolic predictions [139]. Such cultivation approaches will be required to physiologically characterize the so far uncultivated *Nitrospinota* classes. In future studies, the previously successful use of live cell sorting for the cultivation of *Nitrospinia* [12] could be combined with adapted cultivation strategies informed by the genomic potential of other *Nitrospinota* classes that might be able to fix nitrogen and use sulfide or hydrogen as electron donors.

## Supplementary Material

Kop2023_Nitrospinota_ISMEComm_Figures_S1-S10_ycad017

Kop2023_Nitrospinota_ISMEComm_SupplTables_ycad017

Kop2023_Nitrospinota_ISMEComm_SupplText_SupplFigures_REV_ycad017

## Data Availability

All genomes analyzed during this study are listed in Table S1 and are available in the NCBI Genome database [https://www.ncbi.nlm.nih.gov/datasets/genome/].
