# Correction to: Metabolic and phylogenetic diversity in the phylum Nitrospinota revealed by comparative genome analyses

**DOI:** 10.1093/ismeco/ycaf135

**Published:** 2025-09-03

This is a correction to: Linnea F M Kop, Hanna Koch, Mike S M Jetten, Holger Daims, Sebastian Lücker, Metabolic and phylogenetic diversity in the phylum *Nitrospinota* revealed by comparative genome analyses, *ISME Communications*, Volume 4, Issue 1, January 2024, https://doi.org/10.1093/ismeco/ycad017

In the originally published online version of this manuscript, there was an error within Figure 3 (in the arrow for SorAB). Figure 3 should read as follows:


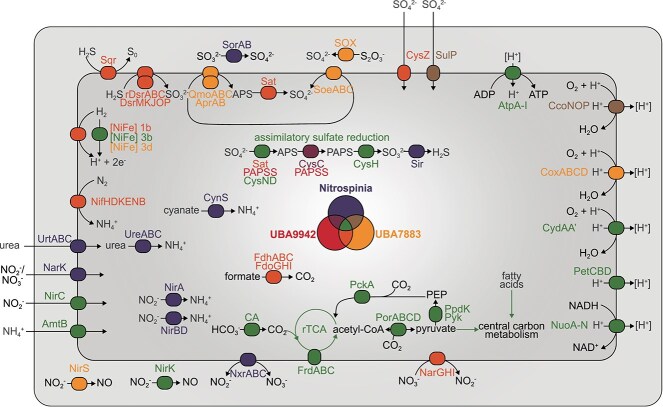


This error has been outlined only in this correction notice to preserve the version of record.

