# Supplementary material for: Metabolic and phylogenetic diversity in the phylum Nitrospinota revealed by comparative genome analyses
Source: ISME Commun. 2024 Jan 10;4(1):ycad017. doi: 10.1093/ismeco/ycad017 (PMC10839748; doi:10.1093/ismeco/ycad017)
Supplement: Kop2023_Nitrospinota_ISMEComm_Figures_S1-S10_ycad017 [file kop2023_nitrospinota_ismecomm_figures_s1-s10_ycad017.pdf]

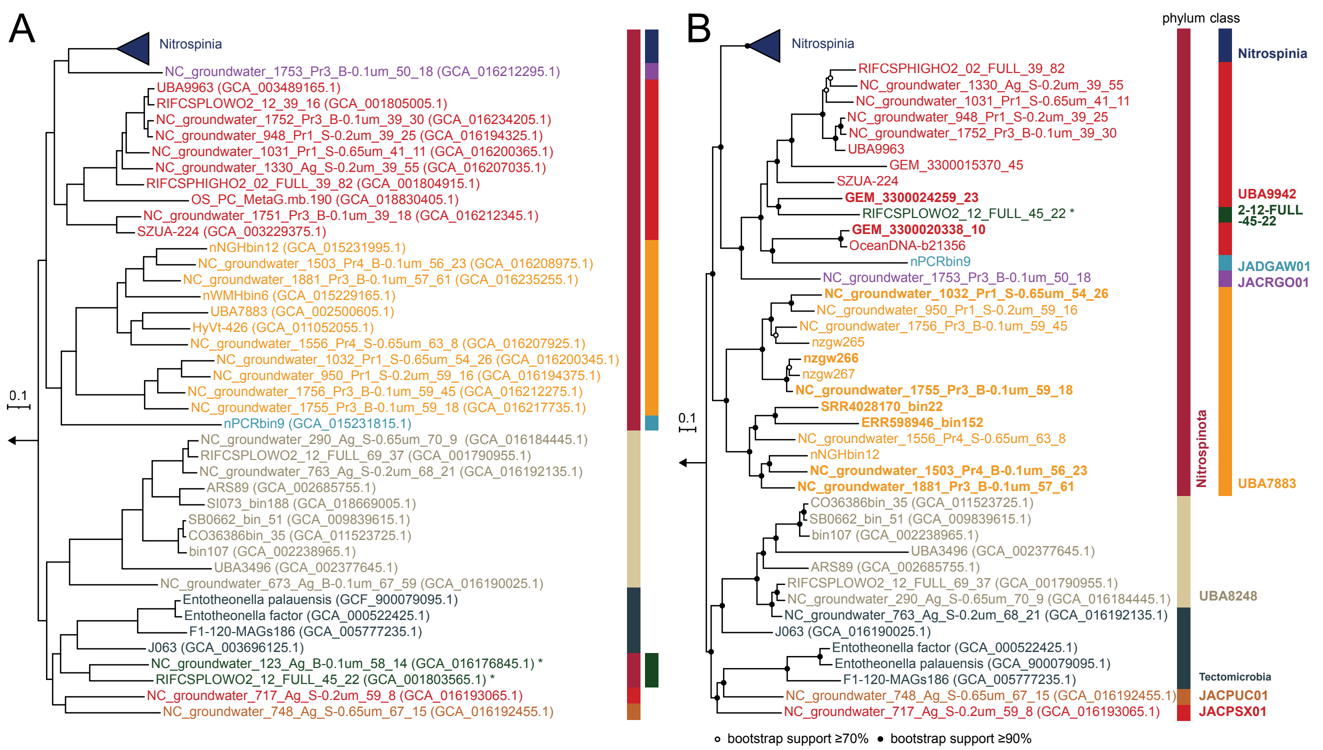

A
Nitrospinia
NC_groundwater_1753_Pr3_B-0.1um_50_18 (GCA_016212295.1)
UBA9963 (GCA_003489165.1)
RIFCSPLOWO2_12_39_16 (GCA_001805005.1)
NC_groundwater_1752_Pr3_B-0.1um_39_30 (GCA_016234205.1)
NC_groundwater_948_Pr1_S-0.2um_39_25 (GCA_016194325.1)
NC_groundwater_1031_Pr1_S-0.65um_41_11 (GCA_016200365.1)
NC_groundwater_1330_Ag_S-0.2um_39_55 (GCA_016207035.1)
RIFCSPHIGHO2_02_FULL_39_82 (GCA_001804915.1)
OS_PC_MetaG.mb.190 (GCA_018830405.1)
NC_groundwater_1751_Pr3_B-0.1um_39_18 (GCA_016212345.1)
SZUA-224 (GCA_003229375.1)
nNGHbin12 (GCA_015231995.1)
NC_groundwater_1503_Pr4_B-0.1um_56_23 (GCA_016208975.1)
NC_groundwater_1881_Pr3_B-0.1um_57_61 (GCA_016235255.1)
nWMHbin6 (GCA_015229165.1)
UBA7883 (GCA_002500605.1)
HyVt-426 (GCA_011052055.1)
NC_groundwater_1556_Pr4_S-0.65um_63_8 (GCA_016207925.1)
NC_groundwater_1032_Pr1_S-0.65um_54_26 (GCA_016200345.1)
NC_groundwater_950_Pr1_S-0.2um_59_16 (GCA_016194375.1)
NC_groundwater_1756_Pr3_B-0.1um_59_45 (GCA_016212275.1)
NC_groundwater_1755_Pr3_B-0.1um_59_18 (GCA_016217735.1)
0.1
nPCRbin9 (GCA_015231815.1)
NC_groundwater_290_Ag_S-0.65um_70_9 (GCA_016184445.1)
RIFCSPLOWO2_12_FULL_69_37 (GCA_001790955.1)
NC_groundwater_763_Ag_S-0.2um_68_21 (GCA_016192135.1)
ARS89 (GCA_002685755.1)
SI073_bin188 (GCA_018669005.1)
SB0662_bin_51 (GCA_009839615.1)
CO36386bin_35 (GCA_011523725.1)
bin107 (GCA_002238965.1)
UBA3496 (GCA_002377645.1)
NC_groundwater_673_Ag_B-0.1um_67_59 (GCA_016190025.1)
Entotheonella palauensis (GCF_900079095.1)
Entotheonella factor (GCA_000522425.1)
F1-120-MAGs186 (GCA_005777235.1)
J063 (GCA_003696125.1)
NC_groundwater_123_Ag_B-0.1um_58_14 (GCA_016176845.1) *
RIFCSPLOWO2_12_FULL_45_22 (GCA_001803565.1) *
NC_groundwater_717_Ag_S-0.2um_59_8 (GCA_016193065.1)
NC_groundwater_748_Ag_S-0.65um_67_15 (GCA_016192455.1)
B
phylum class
Nitrospinia
RIFCSPHIGHO2_02_FULL_39_82
NC_groundwater_1330_Ag_S-0.2um_39_55
NC_groundwater_1031_Pr1_S-0.65um_41_11
NC_groundwater_948_Pr1_S-0.2um_39_25
NC_groundwater_1752_Pr3_B-0.1um_39_30
UBA9963
GEM_3300015370_45
SZUA-224
GEM_3300024259_23
RIFCSPLOWO2_12_FULL_45_22 *
GEM_3300020338_10
OceanDNA-b21356
nPCRbin9
NC_groundwater_1753_Pr3_B-0.1um_50_18
NC_groundwater_1032_Pr1_S-0.65um_54_26
NC_groundwater_950_Pr1_S-0.2um_59_16
NC_groundwater_1756_Pr3_B-0.1um_59_45
nzgw265
nzgw266
nzgw267
NC_groundwater_1755_Pr3_B-0.1um_59_18
SRR4028170_bin22
ERR598946_bin152
NC_groundwater_1556_Pr4_S-0.65um_63_8
nNGHbin12
NC_groundwater_1503_Pr4_B-0.1um_56_23
NC_groundwater_1881_Pr3_B-0.1um_57_61
0.1
CO36386bin_35 (GCA_011523725.1)
SB0662_bin_51 (GCA_009839615.1)
bin107 (GCA_002238965.1)
UBA3496 (GCA_002377645.1)
ARS89 (GCA_002685755.1)
RIFCSPLOWO2_12_FULL_69_37 (GCA_001790955.1)
NC_groundwater_290_Ag_S-0.65um_70_9 (GCA_016184445.1)
NC_groundwater_763_Ag_S-0.2um_68_21 (GCA_016192135.1)
J063 (GCA_016190025.1)
Entotheonella factor (GCA_000522425.1)
Entotheonella palauensis (GCA_900079095.1)
F1-120-MAGs186 (GCA_005777235.1)
NC_groundwater_748_Ag_S-0.65um_67_15 (GCA_016192455.1)
NC_groundwater_717_Ag_S-0.2um_59_8 (GCA_016193065.1)
Nitrospinota
UBA9942
2-12-FULL -45-22
JADGAW01
JACRGO01
UBA7883
UBA8248
Tectomicrobia
JACPUC01
JACPSX01
○ bootstrap support ≥70% ● bootstrap support ≥90%

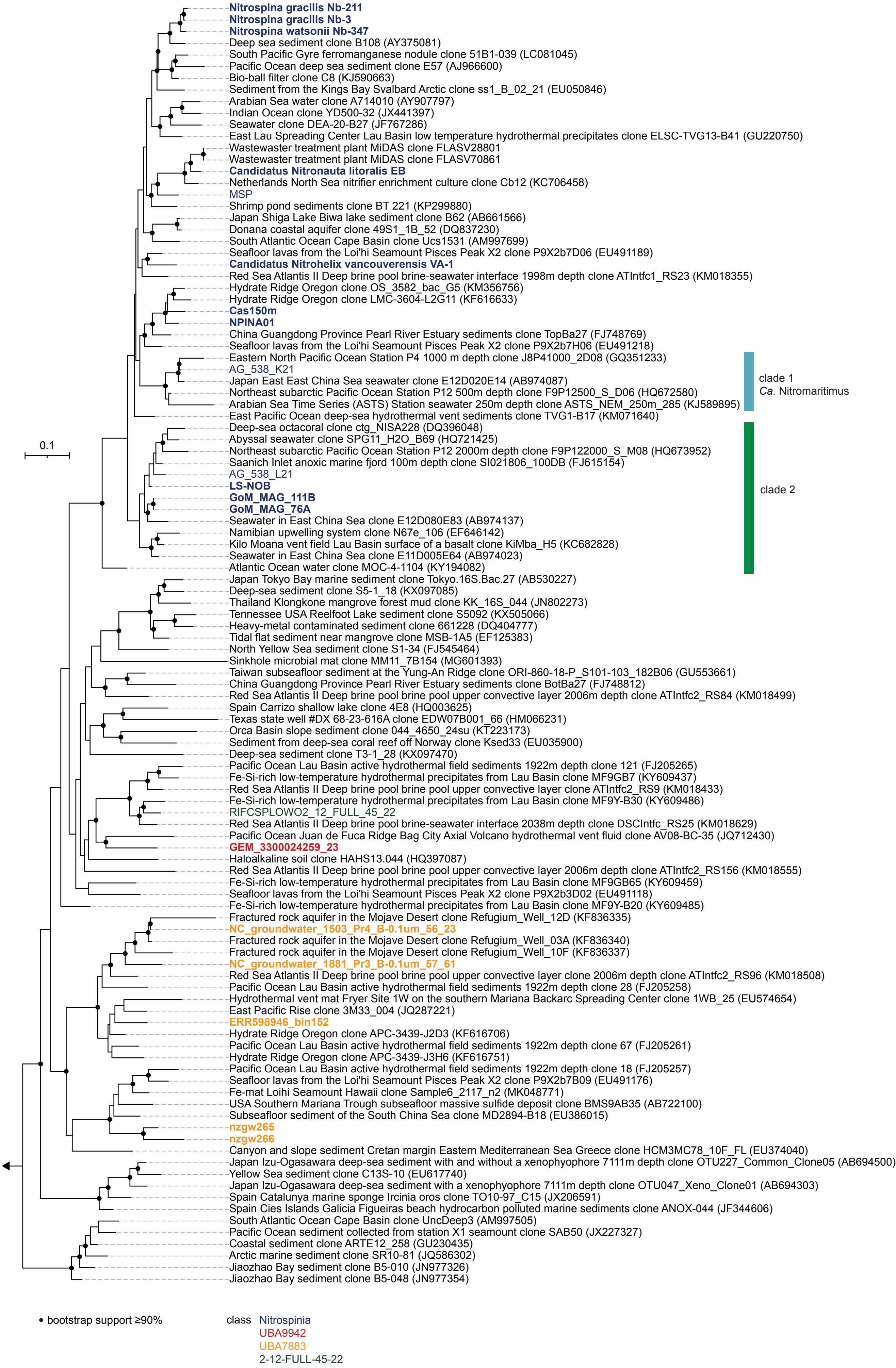

Nitrospina gracilis Nb-211
Nitrospina gracilis Nb-3
Nitrospina watsonii Nb-347
Deep sea sediment clone B108 (AY375081)
South Pacific Gyre ferromanganese nodule clone 51B1-039 (LC081045)
Pacific Ocean deep sea sediment clone E57 (AJ966600)
Bio-ball filter clone C8 (KJ590663)
Sediment from the Kings Bay Svalbard Arctic clone ss1_B_02_21 (EU050846)
Arabian Sea water clone A714010 (AY907797)
Indian Ocean clone YD500-32 (JX441397)
Seawater clone DEA-20-B27 (JF767286)
East Lau Spreading Center Lau Basin low temperature hydrothermal precipitates clone ELSC-TVG13-B41 (GU220750)
Wastewater treatment plant MiDAS clone FLASV28801
Wastewater treatment plant MiDAS clone FLASV70861
Candidatus Nitronauta litoralis EB
Netherlands North Sea nitrifier enrichment culture clone Cb12 (KC706458)
MSP
Shrimp pond sediments clone BT 221 (KP299880)
Japan Shiga Lake Biwa lake sediment clone B62 (AB661566)
Donana coastal aquifer clone 49S1_1B_52 (DQ837230)
South Atlantic Ocean Cape Basin clone Ucs1531 (AM997699)
Seafloor lavas from the Loi'hi Seamount Pisces Peak X2 clone P9X2b7D06 (EU491189)
Candidatus Nitrohelix vancouverensis VA-1
Red Sea Atlantis II Deep brine pool brine-seawater interface 1998m depth clone ATIntfc1_RS23 (KM018355)
Hydrate Ridge Oregon clone OS_3582_bac_G5 (KM356756)
Hydrate Ridge Oregon clone LMC-3604-L2G11 (KF616633)
Cas150m
NPINA01
China Guangdong Province Pearl River Estuary sediments clone TopBa27 (FJ748769)
Seafloor lavas from the Loi'hi Seamount Pisces Peak X2 clone P9X2b7H06 (EU491218)
Eastern North Pacific Ocean Station P4 1000 m depth clone J8P41000_2D08 (GQ351233)
AG_538_K21
Japan East East China Sea seawater clone E12D020E14 (AB974087)
Northeast subarctic Pacific Ocean Station P12 500m depth clone F9P12500_S_D06 (HQ672580)
Arabian Sea Time Series (ASTS) Station seawater 250m depth clone ASTS_NEM_250m_285 (KJ589895)
East Pacific Ocean deep-sea hydrothermal vent sediments clone TVG1-B17 (KM071640)
clade 1
Ca. Nitromaritimus
0.1
Deep-sea octacoral clone ctg_NISA228 (DQ396048)
Abyssal seawater clone SPG11_H2O_B69 (HQ721425)
Northeast subarctic Pacific Ocean Station P12 2000m depth clone F9P122000_S_M08 (HQ673952)
Saanich Inlet anoxic marine fjord 100m depth clone SI021806_100DB (FJ615154)
AG_538_L21
LS-NOB
GoM_MAG_111B
GoM_MAG_76A
Seawater in East China Sea clone E12D080E83 (AB974137)
Namibian upwelling system clone N67e_106 (EF646142)
Kilo Moana vent field Lau Basin surface of a basalt clone KiMba_H5 (KC682828)
Seawater in East China Sea clone E11D005E64 (AB974023)
Atlantic Ocean water clone MOC-4-1104 (KY194082)
clade 2
Japan Tokyo Bay marine sediment clone Tokyo.16S.Bac.27 (AB530227)
Deep-sea sediment clone S5-1_18 (KX097085)
Thailand Klongkone mangrove forest mud clone KK_16S_044 (JN802273)
Tennessee USA Reelfoot Lake sediment clone S5092 (KX505066)
Heavy-metal contaminated sediment clone 661228 (DQ404777)
Tidal flat sediment near mangrove clone MSB-1A5 (EF125383)
North Yellow Sea sediment clone S1-34 (FJ545464)
Sinkhole microbial mat clone MM11_7B154 (MG601393)
Taiwan subseafloor sediment at the Yung-An Ridge clone ORI-860-18-P_S101-103_182B06 (GU553661)
China Guangdong Province Pearl River Estuary sediments clone BotBa27 (FJ748812)
Red Sea Atlantis II Deep brine pool brine pool upper convective layer 2006m depth clone ATIntfc2_RS84 (KM018499)
Spain Carrizo shallow lake clone 4E8 (HQ003625)
Texas state well #DX 68-23-616A clone EDW07B001_66 (HM066231)
Orca Basin slope sediment clone 044_4650_24su (KT223173)
Sediment from deep-sea coral reef off Norway clone Ksed33 (EU035900)
Deep-sea sediment clone T3-1_28 (KX097470)
Pacific Ocean Lau Basin active hydrothermal field sediments 1922m depth clone 121 (FJ205265)
Fe-Si-rich low-temperature hydrothermal precipitates from Lau Basin clone MF9GB7 (KY609437)
Red Sea Atlantis II Deep brine pool brine pool upper convective layer clone ATIntfc2_RS9 (KM018433)
Fe-Si-rich low-temperature hydrothermal precipitates from Lau Basin clone MF9Y-B30 (KY609486)
RIFCSPLOWO2_12_FULL_45_22
Red Sea Atlantis II Deep brine pool brine-seawater interface 2038m depth clone DSCIntfc_RS25 (KM018629)
Pacific Ocean Juan de Fuca Ridge Bag City Axial Volcano hydrothermal vent fluid clone AV08-BC-35 (JQ712430)
GEM_3300024259_23
Haloalkaline soil clone HAHS13.044 (HQ397087)
Red Sea Atlantis II Deep brine pool brine pool upper convective layer 2006m depth clone ATIntfc2_RS156 (KM018555)
Fe-Si-rich low-temperature hydrothermal precipitates from Lau Basin clone MF9GB65 (KY609459)
Seafloor lavas from the Loi'hi Seamount Pisces Peak X2 clone P9X2b3D02 (EU491118)
Fe-Si-rich low-temperature hydrothermal precipitates from Lau Basin clone MF9Y-B20 (KY609485)
Fractured rock aquifer in the Mojave Desert clone Refugium_Well_12D (KF836335)
NC_groundwater_1503_Pr4_B-0.1um_56_23
Fractured rock aquifer in the Mojave Desert clone Refugium_Well_03A (KF836340)
Fractured rock aquifer in the Mojave Desert clone Refugium_Well_10F (KF836337)
NC_groundwater_1881_Pr3_B-0.1um_57_61
Red Sea Atlantis II Deep brine pool brine pool upper convective layer clone 2006m depth clone ATIntfc2_RS96 (KM018508)
Pacific Ocean Lau Basin active hydrothermal field sediments 1922m depth clone 28 (FJ205258)
Hydrothermal vent mat Fryer Site 1W on the southern Mariana Backarc Spreading Center clone 1WB_25 (EU574654)
East Pacific Rise clone 3M33_004 (JQ287221)
ERR598946_bin152
Hydrate Ridge Oregon clone APC-3439-J2D3 (KF616706)
Pacific Ocean Lau Basin active hydrothermal field sediments 1922m depth clone 67 (FJ205261)
Hydrate Ridge Oregon clone APC-3439-J3H6 (KF616751)
Pacific Ocean Lau Basin active hydrothermal field sediments 1922m depth clone 18 (FJ205257)
Seafloor lavas from the Loi'hi Seamount Pisces Peak X2 clone P9X2b7B09 (EU491176)
Fe-mat Loihi Seamount Hawaii clone Sample6_2117_n2 (MK048771)
USA Southern Mariana Trough subseafloor massive sulfide deposit clone BMS9AB35 (AB722100)
Subseafloor sediment of the South China Sea clone MD2894-B18 (EU386015)
nzgw265
nzgw266
Canyon and slope sediment Cretan margin Eastern Mediterranean Sea Greece clone HCM3MC78_10F_FL (EU374040)
Japan Izu-Ogasawara deep-sea sediment with and without a xenophyophore 7111m depth clone OTU227_Common_Clone05 (AB694500)
Yellow Sea sediment clone C13S-10 (EU617740)
Japan Izu-Ogasawara deep-sea sediment with a xenophyophore 7111m depth clone OTU047_Xeno_Clone01 (AB694303)
Spain Catalunya marine sponge Ircinia oros clone TO10-97_C15 (JX206591)
Spain Cies Islands Galicia Figueiras beach hydrocarbon polluted marine sediments clone ANOX-044 (JF344606)
South Atlantic Ocean Cape Basin clone UncDeep3 (AM997505)
Pacific Ocean sediment collected from station X1 seamount clone SAB50 (JX227327)
Coastal sediment clone ARTE12_258 (GU230435)
Arctic marine sediment clone SR10-81 (JQ586302)
Jiaozhao Bay sediment clone B5-010 (JN977326)
Jiaozhao Bay sediment clone B5-048 (JN977354)
• bootstrap support ≥90%
class
Nitrospinia
UBA9942
UBA7883
2-12-FULL-45-22

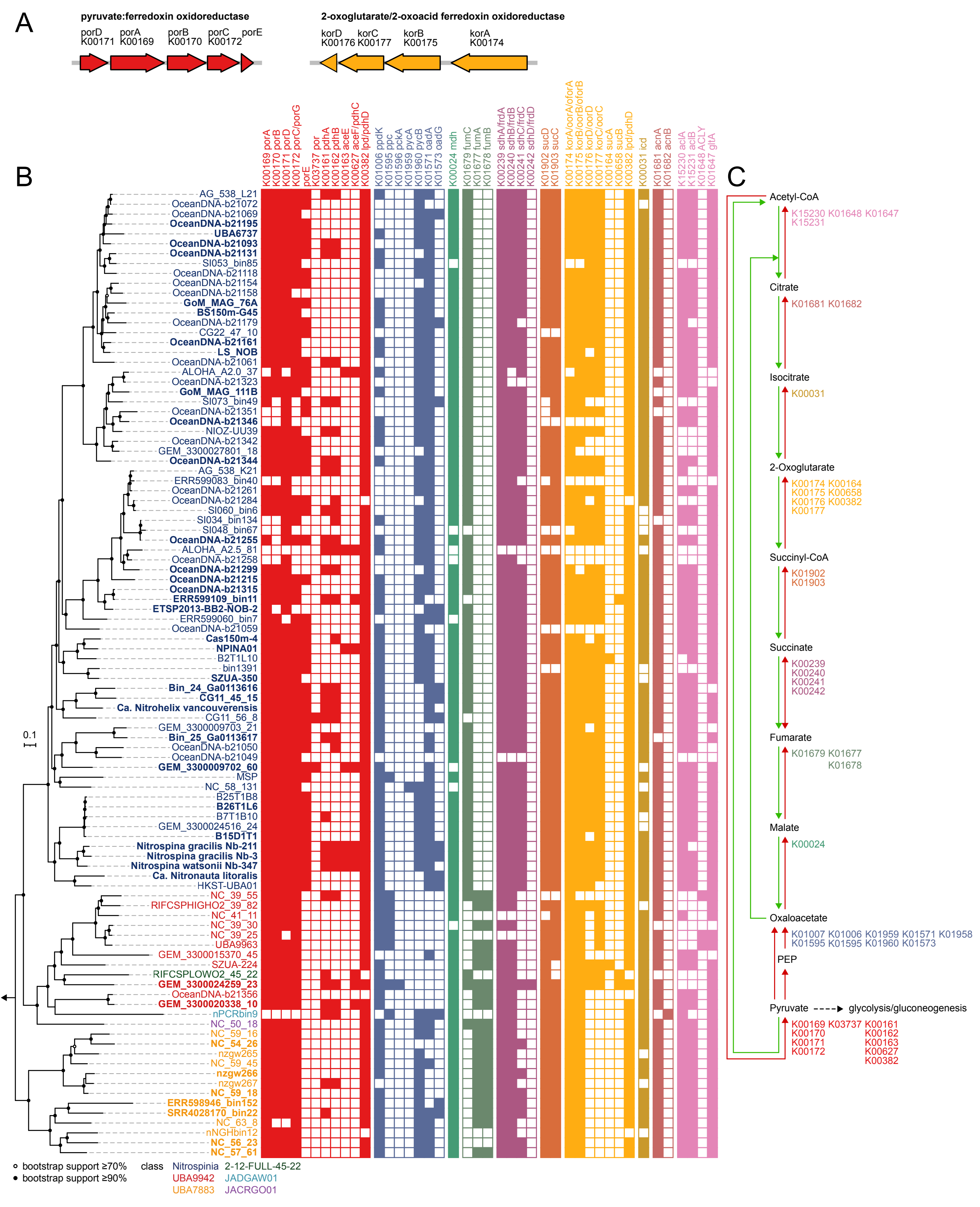

A
pyruvate:ferredoxin oxidoreductase
porD K00171
porA K00169
porB K00170
porC K00172
porE
2-oxoglutarate/2-oxoacid ferredoxin oxidoreductase
korD K00176
korC K00177
korB K00175
korA K00174
B
C
Acetyl-CoA
K15230 K01648 K01647
K15231
Citrate
K01681 K01682
Isocitrate
K00031
2-Oxoglutarate
K00174 K00164
K00175 K00658
K00176 K00382
K00177
Succinyl-CoA
K01902
K01903
Succinate
K00239
K00240
K00241
K00242
Fumarate
K01679 K01677
K01678
Malate
K00024
Oxaloacetate
K01007 K01006 K01959 K01571 K01958
K01595 K01595 K01960 K01573
PEP
Pyruvate
glycolysis/gluconeogenesis
K00169 K03737 K00161
K00170 K00162
K00171 K00163
K00172 K00627
K00382
0.1
bootstrap support ≥70%
bootstrap support ≥90%
class
Nitrospinia
2-12-FULL-45-22
UBA9942
JADGAW01
UBA7883
JACRGO01

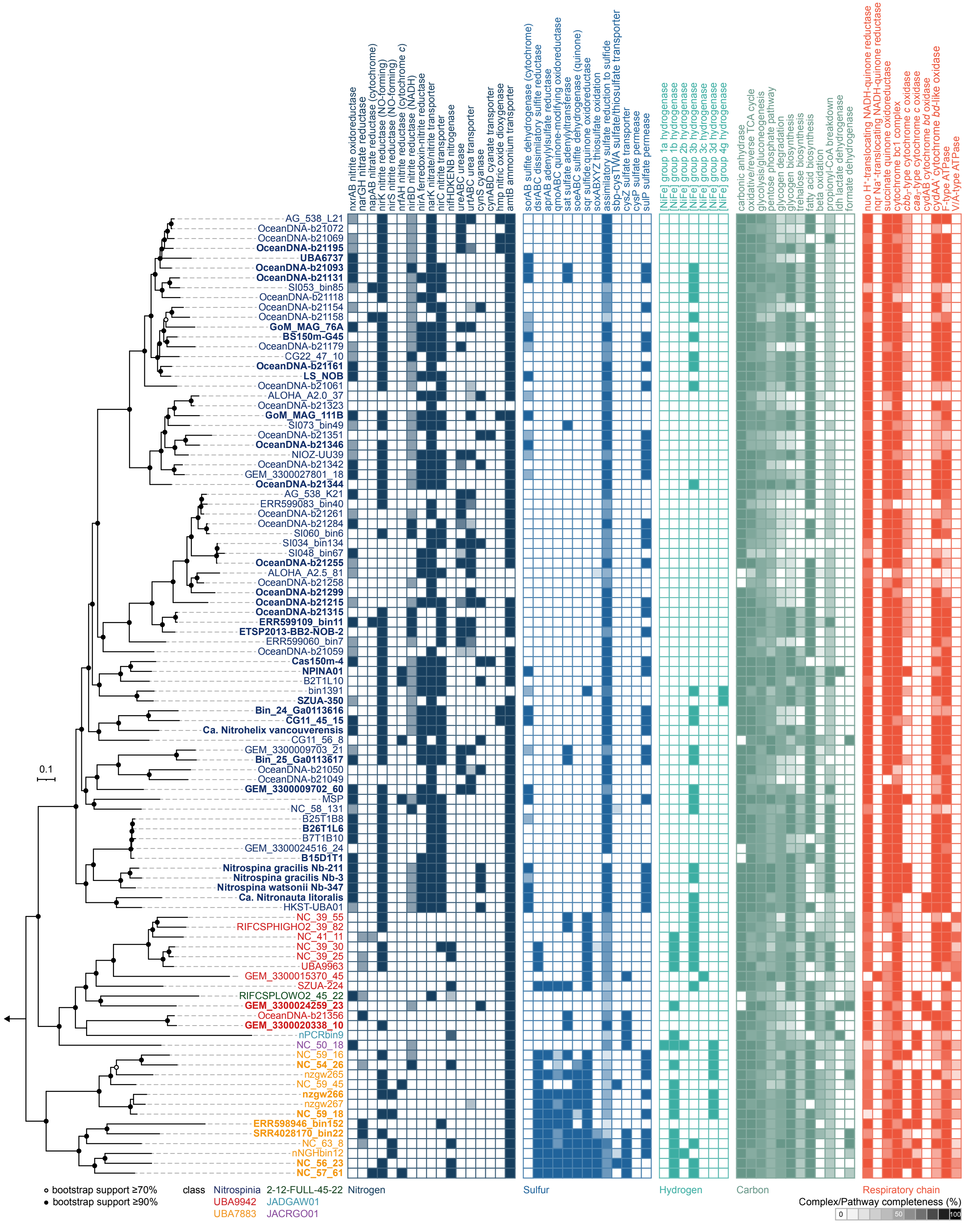

nxrAB nitrite oxidoreductase
narGH nitrate reductase
napAB nitrate reductase (cytochrome)
nirK nitrite reductase (NO-forming)
nirS nitrite reductase (NO-forming)
nrfAH nitrite reductase (cytochrome c)
nirBD nitrite reductase (NADH)
nirA ferredoxin-nitrite reductase
narK nitrate/nitrite transporter
nirC nitrite transporter
nifHDKENB nitrogenase
ureABC urease
urtABC urea transporter
cynS cyanase
cynABD cyanate transporter
hmp nitric oxide dioxygenase
amtB ammonium transporter
sorAB sulfite dehydrogenase (cytochrome)
dsrABC dissimilatory sulfite reductase
aprAB adenylylsulfate reductase
qmoABC quinone-modifying oxidoreductase
sat sulfate adenylyltransferase
soeABC sulfite dehydrogenase (quinone)
sqr sulfide:quinone oxidoreductase
soxABXYZ thiosulfate oxidation
assimilatory sulfate reduction to sulfide
sbp-cysTWA sulfate/thiosulfate transporter
cysZ sulfate transporter
cysP sulfate permease
sulP sulfate permease
[NiFe] group 1a hydrogenase
[NiFe] group 1b hydrogenase
[NiFe] group 2b hydrogenase
[NiFe] group 3b hydrogenase
[NiFe] group 3c hydrogenase
[NiFe] group 3d hydrogenase
[NiFe] group 4g hydrogenase
carbonic anhydrase
oxidative/reverse TCA cycle
glycolysis/gluconeogenesis
pentose phosphate pathway
glycogen degradation
glycogen biosynthesis
trehalose biosynthesis
fatty acid biosynthesis
beta oxidation
propionyl-CoA breakdown
ldh lactate dehydrogenase
formate dehydrogenase
nuo H⁺-translocating NADH-quinone reductase
nqr Na⁺-translocating NADH-quinone reductase
succinate quinone oxidoreductase
cytochrome bc1 complex
cbb3-type cytochrome c oxidase
caa3-type cytochrome c oxidase
cydAB cytochrome bd oxidase
cydAA' cytochrome bd-like oxidase
F-type ATPase
V/A-type ATPase
AG_538_L21
OceanDNA-b21072
OceanDNA-b21069
OceanDNA-b21195
UBA6737
OceanDNA-b21093
OceanDNA-b21131
SI053_bin85
OceanDNA-b21118
OceanDNA-b21154
OceanDNA-b21158
GoM_MAG_76A
BS150m-G45
OceanDNA-b21179
CG22_47_10
OceanDNA-b21161
LS_NOB
OceanDNA-b21061
ALOHA_A2.0_37
OceanDNA-b21323
GoM_MAG_111B
SI073_bin49
OceanDNA-b21351
OceanDNA-b21346
NIOZ-UU39
OceanDNA-b21342
GEM_3300027801_18
OceanDNA-b21344
AG_538_K21
ERR599083_bin40
OceanDNA-b21261
OceanDNA-b21284
SI060_bin6
SI034_bin134
SI048_bin67
OceanDNA-b21255
ALOHA_A2.5_81
OceanDNA-b21258
OceanDNA-b21299
OceanDNA-b21215
OceanDNA-b21315
ERR599109_bin11
ETSP2013-BB2-NOB-2
ERR599060_bin7
OceanDNA-b21059
Cas150m-4
NPINA01
B2T1L10
bin1391
SZUA-350
Bin_24_Ga0113616
CG11_45_15
Ca. Nitrohelix vancouverensis
CG11_56_8
GEM_3300009703_21
Bin_25_Ga0113617
OceanDNA-b21050
OceanDNA-b21049
GEM_3300009702_60
MSP
NC_58_131
B25T1B8
B26T1L6
B7T1B10
GEM_3300024516_24
B15D1T1
Nitrospina gracilis Nb-211
Nitrospina gracilis Nb-3
Nitrospina watsonii Nb-347
Ca. Nitronauta litoralis
HKST-UBA01
NC_39_55
RIFCSPHIGHO2_39_82
NC_41_11
NC_39_30
NC_39_25
UBA9963
GEM_3300015370_45
SZUA-224
RIFCSPLOWO2_45_22
GEM_3300024259_23
OceanDNA-b21356
GEM_3300020338_10
nPCRbin9
NC_50_18
NC_59_16
NC_54_26
nzgw265
NC_59_45
nzgw266
nzgw267
NC_59_18
ERR598946_bin152
SRR4028170_bin22
NC_63_8
nNGHbin12
NC_56_23
NC_57_61
0.1
bootstrap support ≥70%
bootstrap support ≥90%
class
Nitrospinia
2-12-FULL-45-22
UBA9942
JADGAW01
UBA7883
JACRGO01
Nitrogen
Sulfur
Hydrogen
Carbon
Respiratory chain
Complex/Pathway completeness (%)
0
50
100

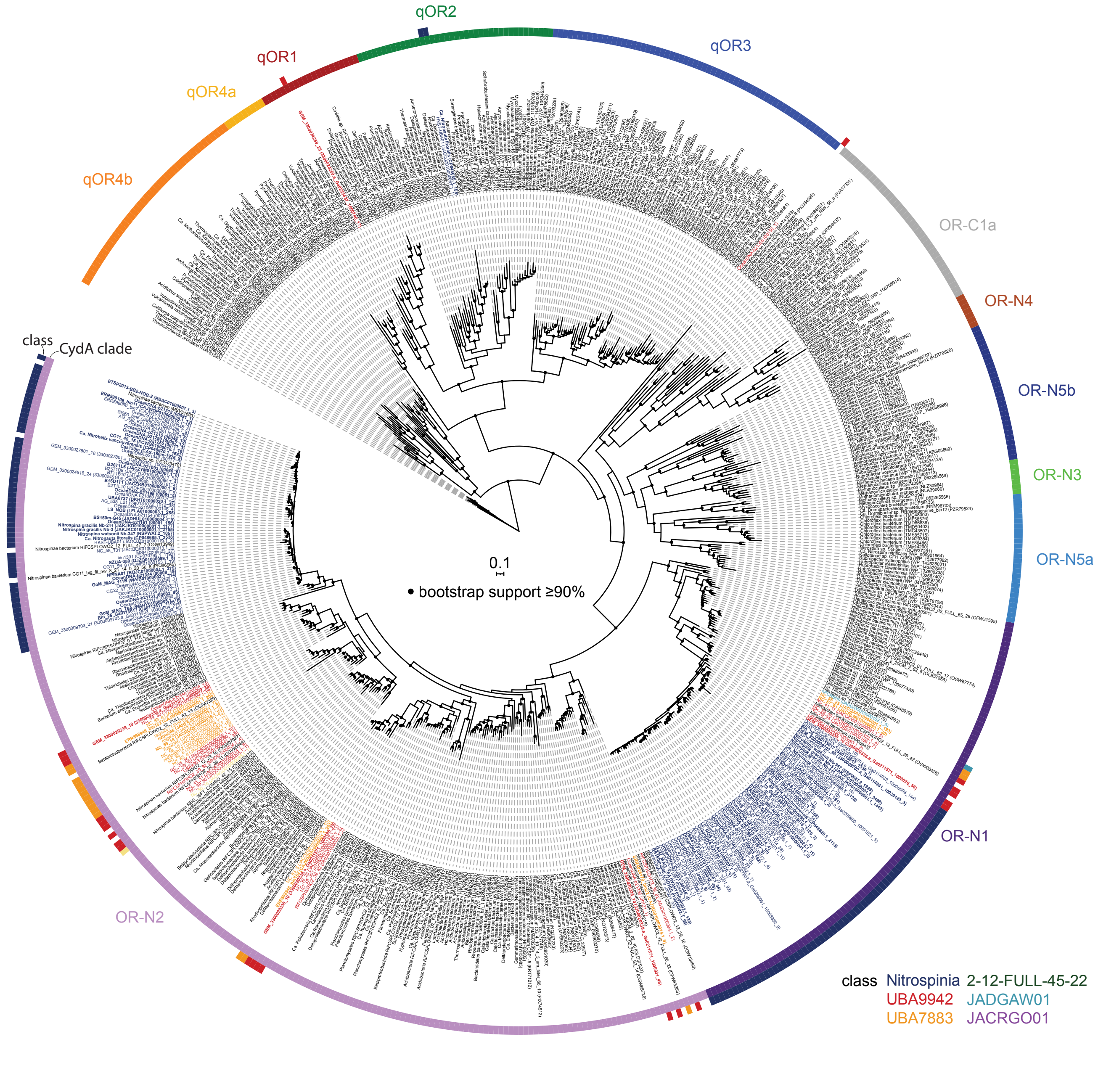

qOR2
qOR3
qOR1
qOR4a
qOR4b
OR-C1a
OR-N4
OR-N5b
OR-N3
OR-N5a
OR-N1
OR-N2
class
CydA clade
0.1
• bootstrap support ≥90%
class Nitrospinia 2-12-FULL-45-22
UBA9942 JADGAW01
UBA7883 JACRGO01

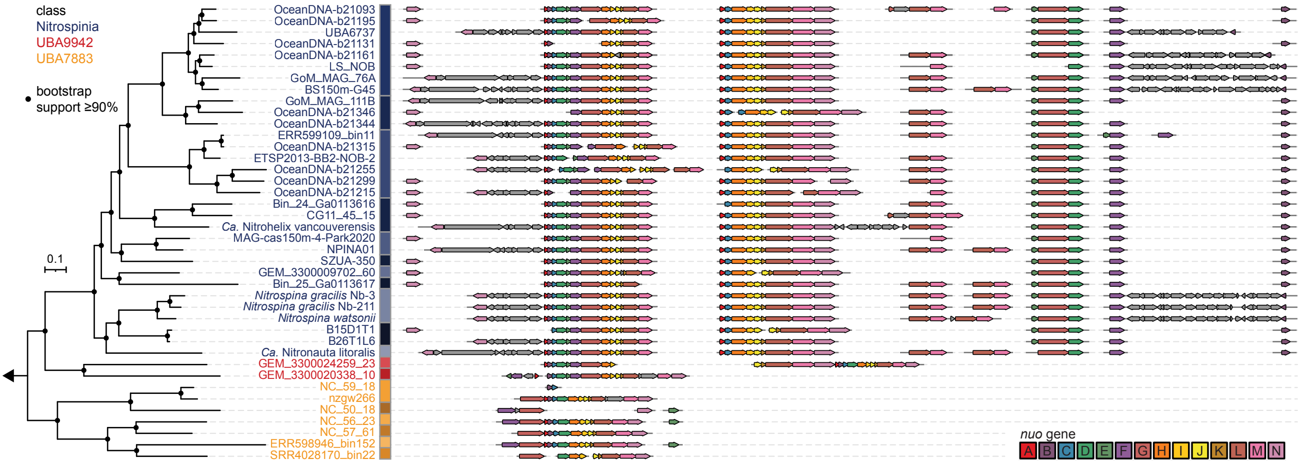
class
Nitrospinia
UBA9942
UBA7883
bootstrap support ≥90%
0.1
OceanDNA-b21093
OceanDNA-b21195
UBA6737
OceanDNA-b21131
OceanDNA-b21161
LS_NOB
GoM_MAG_76A
BS150m-G45
GoM_MAG_111B
OceanDNA-b21346
OceanDNA-b21344
ERR599109_bin11
OceanDNA-b21315
ETSP2013-BB2-NOB-2
OceanDNA-b21255
OceanDNA-b21299
OceanDNA-b21215
Bin_24_Ga0113616
CG11_45_15
Ca. Nitrohelix vancouverensis
MAG-cas150m-4-Park2020
NPINA01
SZUA-350
GEM_3300009702_60
Bin_25_Ga0113617
Nitrospina gracilis Nb-3
Nitrospina gracilis Nb-211
Nitrospina watsonii
B15D1T1
B26T1L6
Ca. Nitronauta litoralis
GEM_3300024259_23
GEM_3300020338_10
NC_59_18
nzgw266
NC_50_18
NC_56_23
NC_57_61
ERR598946_bin152
SRR4028170_bin22
nuo gene
A B C D E F G H I J K L M N

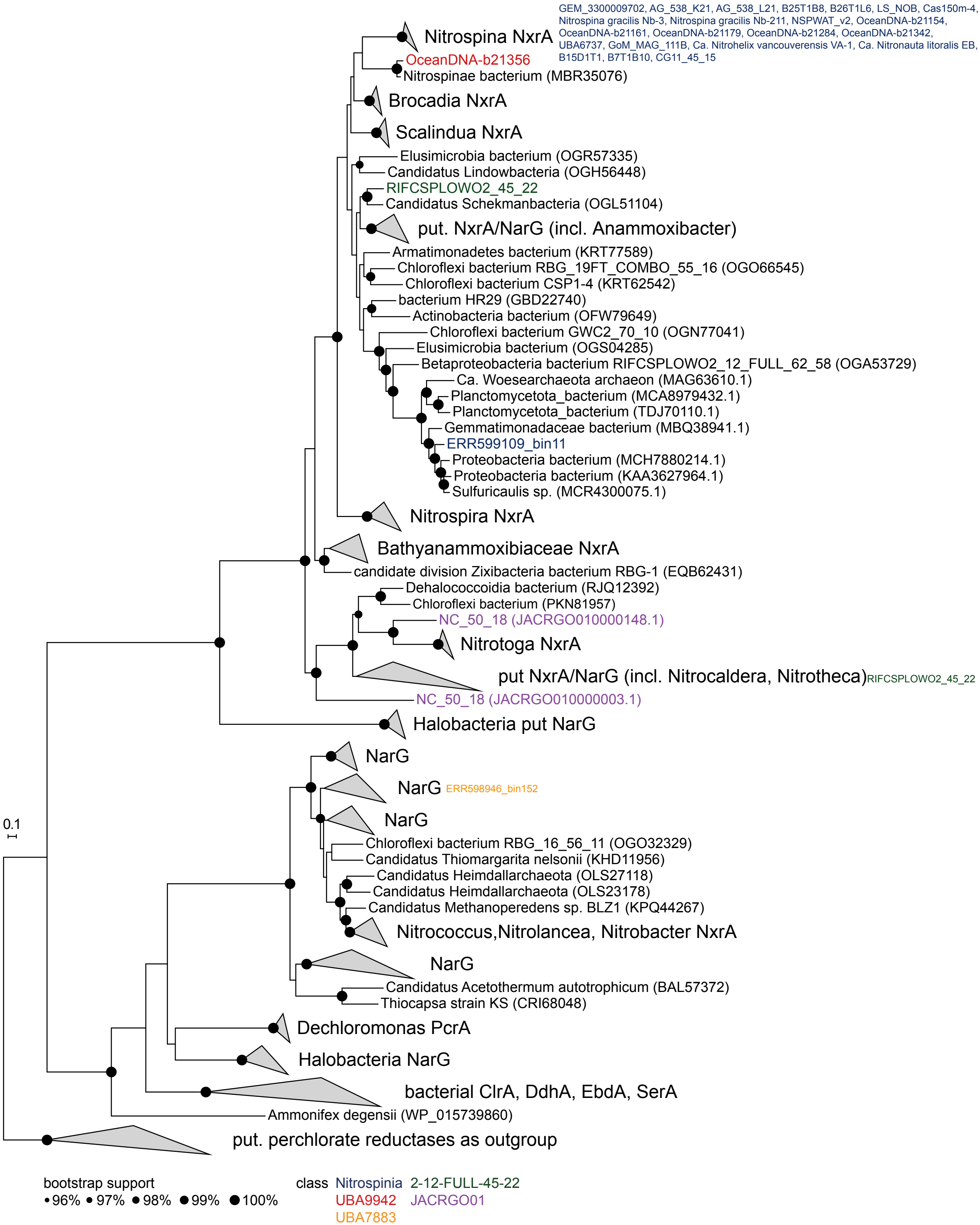
GEM_3300009702, AG_538_K21, AG_538_L21, B25T1B8, B26T1L6, LS_NOB, Cas150m-4, Nitrospina gracilis Nb-3, Nitrospina gracilis Nb-211, NSPWAT_v2, OceanDNA-b21154, OceanDNA-b21161, OceanDNA-b21179, OceanDNA-b21284, OceanDNA-b21342, UBA6737, GoM_MAG_111B, Ca. Nitrohelix vancouverensis VA-1, Ca. Nitronauta litoralis EB, B15D1T1, B7T1B10, CG11_45_15
Nitrospina NxrA
OceanDNA-b21356
Nitrospinae bacterium (MBR35076)
Brocadia NxrA
Scalindua NxrA
Elusimicrobia bacterium (OGR57335)
Candidatus Lindowbacteria (OGH56448)
RIFCSPLOWO2_45_22
Candidatus Schekmanbacteria (OGL51104)
put. NxrA/NarG (incl. Anammoxibacter)
Armatimonadetes bacterium (KRT77589)
Chloroflexi bacterium RBG_19FT_COMBO_55_16 (OGO66545)
Chloroflexi bacterium CSP1-4 (KRT62542)
bacterium HR29 (GBD22740)
Actinobacteria bacterium (OFW79649)
Chloroflexi bacterium GWC2_70_10 (OGN77041)
Elusimicrobia bacterium (OGS04285)
Betaproteobacteria bacterium RIFCSPLOWO2_12_FULL_62_58 (OGA53729)
Ca. Woesearchaeota archaeon (MAG63610.1)
Planctomycetota_bacterium (MCA8979432.1)
Planctomycetota_bacterium (TDJ70110.1)
Gemmatimonadaceae bacterium (MBQ38941.1)
ERR599109_bin11
Proteobacteria bacterium (MCH7880214.1)
Proteobacteria bacterium (KAA3627964.1)
Sulfuricaulis sp. (MCR4300075.1)
Nitrospira NxrA
Bathyanammoxibiaceae NxrA
candidate division Zixibacteria bacterium RBG-1 (EQB62431)
Dehalococcoidia bacterium (RJQ12392)
Chloroflexi bacterium (PKN81957)
NC_50_18 (JACRGO010000148.1)
Nitrotoga NxrA
put NxrA/NarG (incl. Nitrocaldera, Nitrotheca) RIFCSPLOWO2_45_22
NC_50_18 (JACRGO010000003.1)
Halobacteria put NarG
NarG
NarG ERR598946_bin152
NarG
Chloroflexi bacterium RBG_16_56_11 (OGO32329)
Candidatus Thiomargarita nelsonii (KHD11956)
Candidatus Heimdallarchaeota (OLS27118)
Candidatus Heimdallarchaeota (OLS23178)
Candidatus Methanoperedens sp. BLZ1 (KPQ44267)
Nitrococcus,Nitrolancea, Nitrobacter NxrA
NarG
Candidatus Acetothermum autotrophicum (BAL57372)
Thiocapsa strain KS (CRI68048)
Dechloromonas PcrA
Halobacteria NarG
bacterial ClrA, DdhA, EbdA, SerA
Ammonifex degensii (WP_015739860)
put. perchlorate reductases as outgroup
0.1
bootstrap support
96% 97% 98% 99% 100%
class Nitrospinia 2-12-FULL-45-22
UBA9942 JACRGO01
UBA7883

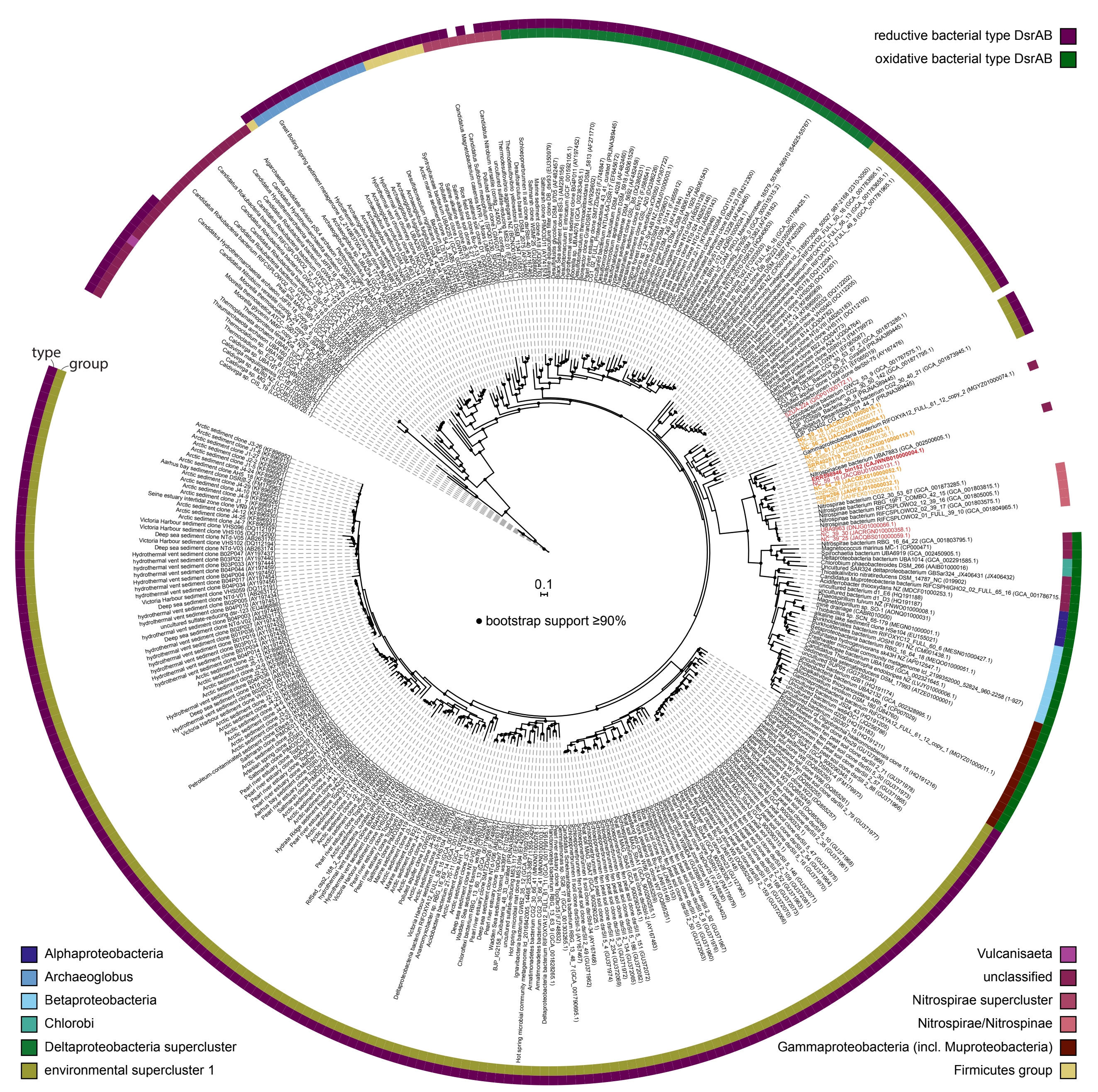
reductive bacterial type DsrAB
oxidative bacterial type DsrAB
type
group
0.1
● bootstrap support ≥90%
Alphaproteobacteria
Archaeoglobus
Betaproteobacteria
Chlorobi
Deltaproteobacteria supercluster
environmental supercluster 1
Vulcanisaeta
unclassified
Nitrospirae supercluster
Nitrospirae/Nitrospinae
Gammaproteobacteria (incl. Muproteobacteria)
Firmicutes group

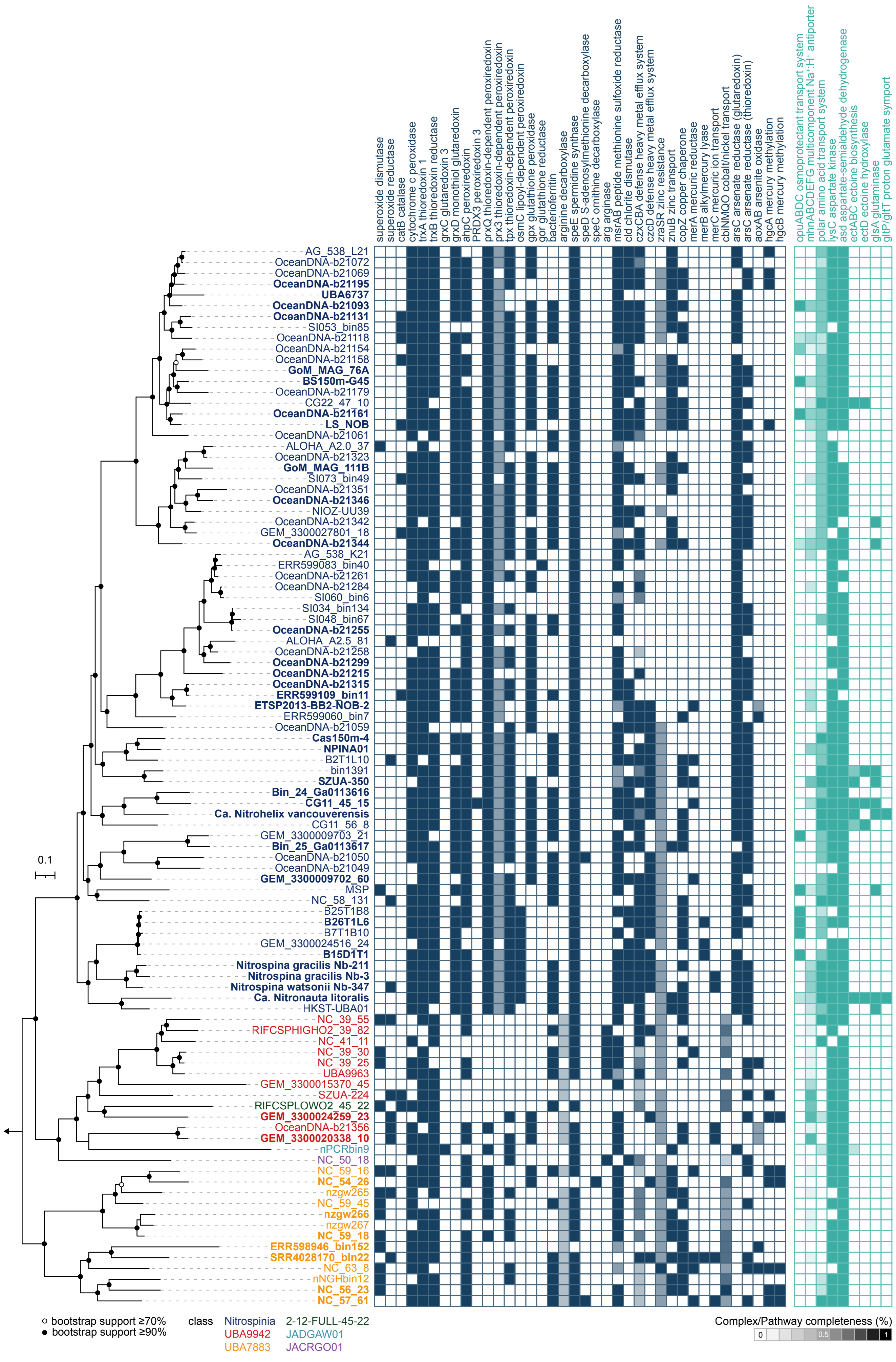

superoxide dismutase
superoxide reductase
catB catalase
cytochrome c peroxidase
trxA thioredoxin 1
trxB thioredoxin reductase
grxC glutaredoxin 3
grxD monothiol glutaredoxin
ahpC peroxiredoxin
PRDX3 peroxiredoxin 3
prxQ thioredoxin-dependent peroxiredoxin
prx3 thioredoxin-dependent peroxiredoxin
tpx thioredoxin-dependent peroxiredoxin
osmC lipoyl-dependent peroxiredoxin
gpx glutathione peroxidase
gor glutathione reductase
bacterioferritin
arginine decarboxylase
speE spermidine synthase
speD S-adenosylmethionine decarboxylase
speC ornithine decarboxylase
arg arginase
msrAB peptide methionine sulfoxide reductase
cld chlorite dismutase
czcCBA defense heavy metal efflux system
czcD defense heavy metal efflux system
zraSR zinc resistance
znuB zinc transport
copZ copper chaperone
merA mercuric reductase
merB alkylmercury lyase
merC mercuric ion transport
cbiNMQO cobalt/nickel transport
arsC arsenate reductase (glutaredoxin)
arsC arsenate reductase (thioredoxin)
aoxAB arsenite oxidase
hgcA mercury methylation
hgcB mercury methylation
opuABDC osmoprotectant transport system
mhnABCDEFG multicomponent Na⁺:H⁺ antiporter
polar amino acid transport system
lysC aspartate kinase
asd aspartate-semialdehyde dehydrogenase
ectABC ectoine biosynthesis
ectD ectoine hydroxylase
glsA glutaminase
gltP/gltT proton glutamate symport
AG_538_L21
OceanDNA-b21072
OceanDNA-b21069
OceanDNA-b21195
UBA6737
OceanDNA-b21093
OceanDNA-b21131
SI053_bin85
OceanDNA-b21118
OceanDNA-b21154
OceanDNA-b21158
GoM_MAG_76A
BS150m-G45
OceanDNA-b21179
CG22_47_10
OceanDNA-b21161
LS_NOB
OceanDNA-b21061
ALOHA_A2.0_37
OceanDNA-b21323
GoM_MAG_111B
SI073_bin49
OceanDNA-b21351
OceanDNA-b21346
NIOZ-UU39
OceanDNA-b21342
GEM_3300027801_18
OceanDNA-b21344
AG_538_K21
ERR599083_bin40
OceanDNA-b21261
OceanDNA-b21284
SI060_bin6
SI034_bin134
SI048_bin67
OceanDNA-b21255
ALOHA_A2.5_81
OceanDNA-b21258
OceanDNA-b21299
OceanDNA-b21215
OceanDNA-b21315
ERR599109_bin11
ETSP2013-BB2-NOB-2
ERR599060_bin7
OceanDNA-b21059
Cas150m-4
NPINA01
B2T1L10
bin1391
SZUA-350
Bin_24_Ga0113616
CG11_45_15
Ca. Nitrohelix vancouverensis
CG11_56_8
GEM_3300009703_21
Bin_25_Ga0113617
OceanDNA-b21050
OceanDNA-b21049
GEM_3300009702_60
MSP
NC_58_131
B25T1B8
B26T1L6
B7T1B10
GEM_3300024516_24
B15D1T1
Nitrospina gracilis Nb-211
Nitrospina gracilis Nb-3
Nitrospina watsonii Nb-347
Ca. Nitronauta litoralis
HKST-UBA01
NC_39_55
RIFCSPHIGHO2_39_82
NC_41_11
NC_39_30
NC_39_25
UBA9963
GEM_3300015370_45
SZUA-224
RIFCSPLOWO2_45_22
GEM_3300024259_23
OceanDNA-b21356
GEM_3300020338_10
nPCRbin9
NC_50_18
NC_59_16
NC_54_26
nzgw265
NC_59_45
nzgw266
nzgw267
NC_59_18
ERR598946_bin152
SRR4028170_bin22
NC_63_8
nNGHbin12
NC_56_23
NC_57_61
0.1
○ bootstrap support ≥70%
● bootstrap support ≥90%
class
Nitrospinia
UBA9942
UBA7883
2-12-FULL-45-22
JADGAW01
JACRGO01
Complex/Pathway completeness (%)
0
0.5
1

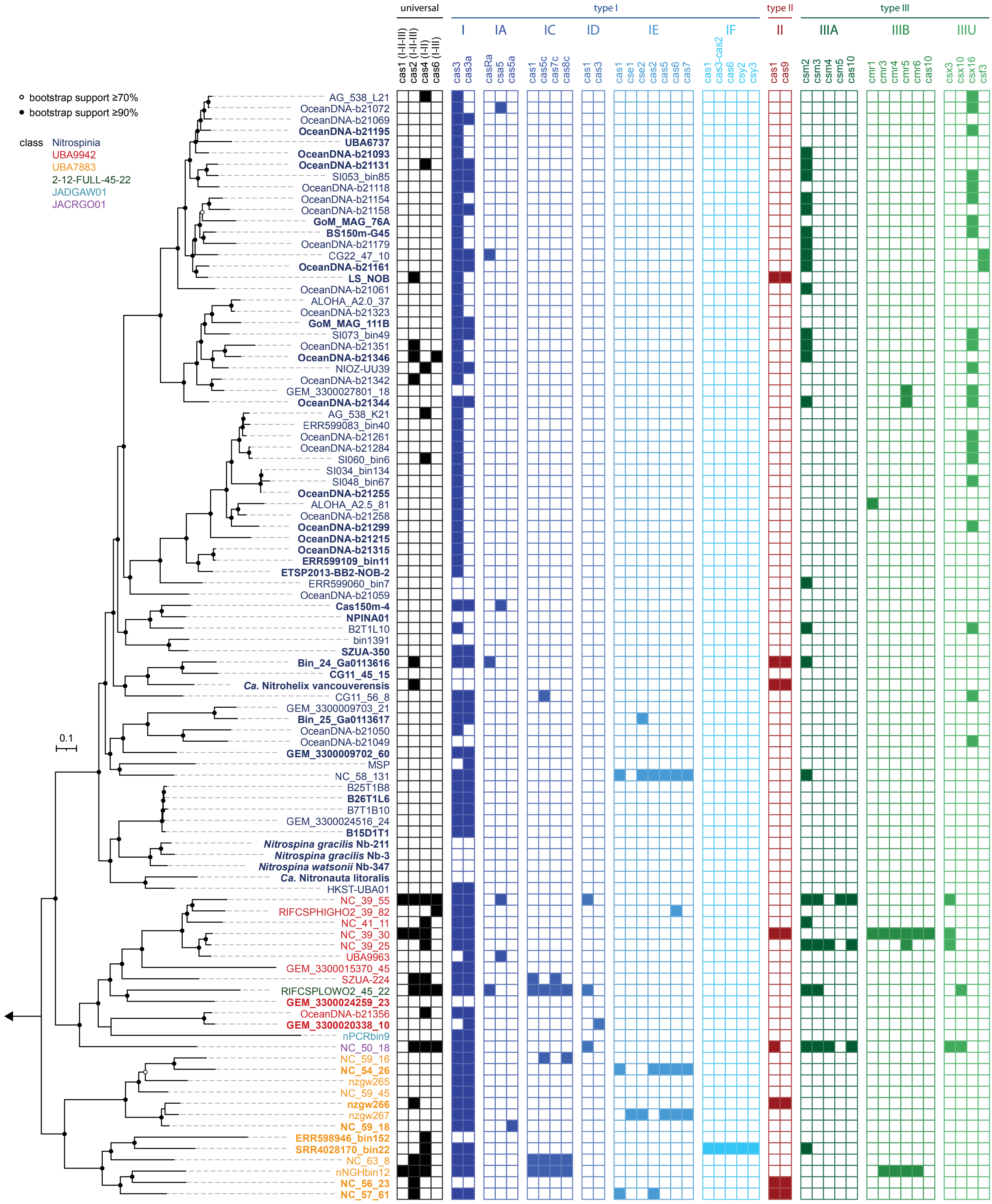

universal
type I
type II
type III
I
IA
IC
ID
IE
IF
II
IIIA
IIIB
IIIU
cas1 (I-II-III)
cas2 (I-II-III)
cas4 (I-II)
cas6 (I-III)
cas3
cas3a
casRa
cas5
cas5a
cas1
cas5c
cas7c
cas8c
cas1
cas3
cas1
cse1
cse2
cas2
cas5
cas6
cas7
cas1
cas3-cas2
cas6
csy2
csy3
cas1
cas9
csm2
csm3
csm4
csm5
cas10
cmr1
cmr3
cmr4
cmr5
cmr6
cas10
csx3
csx10
csx16
csf3
bootstrap support ≥70%
bootstrap support ≥90%
class
Nitrospinia
UBA9942
UBA7883
2-12-FULL-45-22
JADGAW01
JACRGO01
0.1
AG_538_L21
OceanDNA-b21072
OceanDNA-b21069
OceanDNA-b21195
UBA6737
OceanDNA-b21093
OceanDNA-b21131
SI053_bin85
OceanDNA-b21118
OceanDNA-b21154
OceanDNA-b21158
GoM_MAG_76A
BS150m-G45
OceanDNA-b21179
CG22_47_10
OceanDNA-b21161
LS_NOB
OceanDNA-b21061
ALOHA_A2.0_37
OceanDNA-b21323
GoM_MAG_111B
SI073_bin49
OceanDNA-b21351
OceanDNA-b21346
NIOZ-UU39
OceanDNA-b21342
GEM_3300027801_18
OceanDNA-b21344
AG_538_K21
ERR599083_bin40
OceanDNA-b21261
OceanDNA-b21284
SI060_bin6
SI034_bin134
SI048_bin67
OceanDNA-b21255
ALOHA_A2.5_81
OceanDNA-b21258
OceanDNA-b21299
OceanDNA-b21215
OceanDNA-b21315
ERR599109_bin11
ETSP2013-BB2-NOB-2
ERR599060_bin7
OceanDNA-b21059
Cas150m-4
NPINA01
B2T1L10
bin1391
SZUA-350
Bin_24_Ga0113616
CG11_45_15
Ca. Nitrohelix vancouverensis
CG11_56_8
GEM_3300009703_21
Bin_25_Ga0113617
OceanDNA-b21050
OceanDNA-b21049
GEM_3300009702_60
MSP
NC_58_131
B25T1B8
B26T1L6
B7T1B10
GEM_3300024516_24
B15D1T1
Nitrospina gracilis Nb-211
Nitrospina gracilis Nb-3
Nitrospina watsonii Nb-347
Ca. Nitronauta litoralis
HKST-UBA01
NC_39_55
RIFCSPHIGHO2_39_82
NC_41_11
NC_39_30
NC_39_25
UBA9963
GEM_3300015370_45
SZUA-224
RIFCSPLOWO2_45_22
GEM_3300024259_23
OceanDNA-b21356
GEM_3300020338_10
nPCRbin9
NC_50_18
NC_59_16
NC_54_26
nzgw265
NC_59_45
nzgw266
nzgw267
NC_59_18
ERR598946_bin152
SRR4028170_bin22
NC_63_8
nNGHbin12
NC_56_23
NC_57_61
