# Supplementary material for: Metabolic and phylogenetic diversity in the phylum Nitrospinota revealed by comparative genome analyses
Source: ISME Commun. 2024 Jan 10;4(1):ycad017. doi: 10.1093/ismeco/ycad017 (PMC10839748; doi:10.1093/ismeco/ycad017)
Supplement: Kop2023_Nitrospinota_ISMEComm_SupplText_SupplFigures_REV_ycad017 [file kop2023_nitrospinota_ismecomm_suppltext_supplfigures_rev_ycad017.docx]

Metabolic and phylogenetic diversity in the phylum *Nitrospinota* revealed by comparative genome analyses

Linnea F.M. Kop^1,2^, Hanna Koch^1,#^, Mike S.M. Jetten^1^, Holger Daims^2^, Sebastian Lücker^1,*^

^1^ Department of Microbiology, Radboud Institute for Biological and Environmental Sciences, Radboud University, Heyendaalseweg 135, 6525 AJ, Nijmegen, the Netherlands

^2^ Division of Microbial Ecology, Centre for Microbiology and Environmental Systems Science, University of Vienna, Djerassiplatz 1, 1030 Vienna, Austria

^3^ Bioresources Unit, Center for Health & Bioresources, AIT Austrian Institute of Technology GmbH, Konrad-Lorenz-Straße 24, 3430 Tulln an der Donau, Austria

*Corresponding author: Sebastian Lücker, Department of Microbiology, Radboud Institute for Biological and Environmental Sciences, Radboud University, Heyendaalseweg 135, 6525 AJ, Nijmegen, The Netherlands, s.luecker@science.ru.nl

# **Supplementary Materials**

Contains supplementary text and supplementary figures S1-S10. Supplementary tables S1-S7 are provided in a separate Excel file.

## Supplementary Text

*Respiratory Chain*

In the class *Nitrospinia*, the NUO-1 genes encoding complex I of the respiratory chain are clustered in the order *nuoABCDEFGHIJKLM* with the *nuoN* gene located several genes upstream (Fig. S6). In contrast, the genes encoding the alternative complex NUO-2 are spread across the genome, with *nuoACHIJKLMN* and *nuoEDG* forming separate clusters, while *nuoB* and *nuoF* are usually found at different locations elsewhere in the genome. Furthermore, most genomes contain additional copies of the *nuoLM* genes. In the two high-quality MAGs belonging to the class UBA9942, the *nuo* genes are found in different orders. While GEM_3300020338_10 has duplicated *nuoF* and *nuoB* genes, GEM_3300024259_23 contains all genes twice except for the *nuoEFG* genes. In the class UBA7883 MAGs, only one set of the complex I genes is present in the order *nuoFGABCDHIJKLMN* with a *nuoE* in another location. Notably, not all genes could be found in all MAGs, which may, however, be a result of genome incompleteness (Fig. S6).

Complex II of the respiratory chain, the succinate:quinone oxidoreductase (SQOR), catalyzes the oxidation of succinate to fumarate or the opposite reaction of fumarate reduction coupled to quinol oxidation [1]. Based on subunit composition and structure, the SQORs can be categorized into five different types (A-E). Most analyzed *Nitrospinia* MAGs contain both type B as well as type E SQORs. MAGs belonging to the class UBA7883 encode only a type E SQOR, and class UBA9942 MAGs contain either a type B or a type E SQOR.

Complex III, the quinol:cytochrome *c* oxidoreductase, is found in all analyzed *Nitrospinota* genomes. Almost all the genomes encode a cytochrome *b* subunit that is split into two separate proteins (*petB* and *petD*), analogous to cytochrome *b*_6_ in chloroplasts and cyanobacteria, next to a Rieske [2Fe-2S] subunit (*petC*) and a multiheme cytochrome *c*. Furthermore, elsewhere in the genome, most genomes contain a second, unsplit copy of the cytochrome *b* subunit (Table S4).

It has been hypothesized that under anoxic conditions, NOB may use chlorate as an electron acceptor [2, 3]. The produced chlorite may be converted into chloride and O_2_ by a chlorite dismutase (Cld) [4, 5], which is encoded in most class *Nitrospinia* genomes. However, experimental evidence for in vivo O_2_ production and activity or even growth by chlorate reduction is lacking.

*Nutrient import*

For phosphate import, the high-affinity phosphate transporter (PstSCABU) and phosphonate transport system (PhnDEC) are encoded in most of the *Nitrospinota* genomes. These transporters enable the organisms to obtain scarce nutrients, as has previously been described for *Ca.* Nitromaritima RS [6]. In addition to phosphorus, several genomes also encode proteins for iron acquisition (Table S6). Iron uptake is important for NOB due to their high demand for iron as a cofactor of cytochromes and ferredoxins, which are all essential for their core metabolism. Next to iron, the NXR also requires molybdenum as a cofactor and the molybdate transport system *modABC* is found in the majority of the *Nitrospinota* genomes.

*Motility*

The genetic potential for motility is present in most *Nitrospinota* genomes, as they encode genes for flagella and chemotaxis. While motility has not been observed in pure *Nitrospina* cultures [7, 8], these traits may be widespread in marine nitrite oxidizers. A recent study found that some *Nitrospinota* encode proteins involved in magnetotaxis [9], which could enable them to move in response to the Earth’s geomagnetic field [10]. Correspondingly, genes involved in magnetosome formation were identified by FeGenie in a few of the genomes (Bin_25_Ga0113617, GEM_3300027801_18 and SI053_bin85 [class *Nitrospinia*], NC_50_18 [class JACRGO01], nNGHbin12 and SRR4028170_bin22 [class UBA7883]) (Table S6).

*Stress Defense and Resistance*

Most *Nitrospinia* MAGs lack classical defense mechanisms against reactive oxygen species (ROS) such as a superoxide dismutase for the disproportionation of superoxide (O_2_^-^) into O_2_ and hydrogen peroxide (H_2_O_2_) (Fig. S9). Few of the analyzed *Nitrospinia* genomes encode a superoxide reductase for the conversion of O_2_^-^ to H_2_O_2_, and OceanDNA-b21131, LS_NOB and ERR599109_bin11 are the only *Nitrospinia* bins encoding a catalase for breaking down H_2_O_2_ into water and O_2_. In contrast, many of the members of the classes UBA9942 and UBA7883 encode a superoxide dismutase from the iron-manganese family or a superoxide reductase. As described for the cultured *Nitrospinia* [11–13], *Nitrospinota* genomes contain genes encoding a cytochrome *c* peroxidase and various glutaredoxin and several thioredoxin reductases. Furthermore, many of the MAGs contain a glutathione peroxidase and bacterioferritin, which also play a role in the oxidative stress response of bacteria [14, 15]. Furthermore, polyamines could contribute to ROS defense as most *Nitrospinota* MAGs encode spermidine synthase. Lastly, almost all of the genomes encode the peptide methionine sulfoxide reductase (Fig. S9), which can repair ROS damage to methionine residues [16].

In addition to ROS defense, *Nitrospinota* are equipped with several proteins involved in defense against toxic metals. Almost all class *Nitrospinia* genomes encode a glutaredoxin or thioredoxin-dependent arsenate reductase (ArsC; K00537 and K03741), or both. While the MAG nPCRbin9 of class JADGAW01 contains a glutaredoxin-dependent enzyme variant, genomes of the classes UBA9942, UBA7883, and JACRGO01 only encode thioredoxin-dependent arsenate reductases, if any at all. Arsenite oxidase genes (*aoxAB*) that were previously found in *Nitrospinota* genomes [17] could only be identified in few of the MAGs (ETSP2013-BB2-NOB-2 [class *Nitrospinia*], NC_39_25 and OceanDNA-b21356 [class UBA9942], and GEM_3300020338_10, ERR599060_bin7, and ERR598946_bin152 [class UBA7883]) and might play a role in arsenite detoxification to arsenate. A heavy metal efflux system (CzxCBA) was identified in many *Nitrospinota* MAGs and in addition cobalt/nickel transporter genes (*cbiNMQO*) were found in MAGs of class UBA9942 and UBA7883.

Another heavy metal that is of particular concern to human health is methylmercury in the marine environment [18]. *Nitrospina* species have been identified as potential mercury methylators [19–21], and a recent study found that *Nitrospinia* organisms express the mercury methylation gene *hgcA* [22]. This gene was indeed found in four of the *Nitrospinia* genomes, but no *hgcB* sequence could be identified in these MAGs. In contrast, several class UBA7883 and a UBA9942 MAG encode both *hgcA* and *hgcB* (Fig. S9). As the presence of *hgcAB* genes in *Nitrospinota* MAGs alone is insufficient evidence for a potential role in mercury methylation [22], pure cultures of *hgcAB*-encoding *Nitrospinota* would be needed to test their capacity for mercury methylation. Unfortunately, none of these organisms has been cultured so far.

Several genes involved in osmoprotection were found in the *Nitrospinota* genomes, but no universal pathway was identified in all of them (Fig. S9). The Mnh-type Na^+^/H^+^ antiporter probably confers salt tolerance to some *Nitrospinota*, but is not conserved. Notably, these genes are lacking in the class UBA7883 MAGs, many of which were derived from groundwater. In addition, a few *Nitrospinia* genomes encode an osmoprotectant transport system (OpuABDC). The two cultured strains *Ca.* N. vancouverensis and *Ca.* N. litoralis contain the genes necessary for ectoine biosynthesis (EctABC), the ectoine hydroxylase (EctD), glutaminase and a glutamate/H^+^ symporter [12]. However, also these features seem to be restricted to only a few *Nitrospinia* genomes (Fig. S9).

The CRISPR-Cas viral defense system is conserved in most *Nitrospinota*. CRISPR arrays were identified in the MAGs from all analyzed classes except for the MAG nPCRbin9 from class JADGAW01. Like *Ca.* N. vancouverensis, several MAGs encode Cas proteins for the Type II CRISPR-Cas system on the same contig (Table S7) [12], but Type I and Type III Cas proteins were also identified in several MAGs, indicating a broad diversity of Cas systems in *Nitrospinota* (Table S7, Fig. S10). The presence of diverse CRISPR-Cas systems in several of the *Nitrospinota* genomes is in line with the hypothesis proposed by Kitzinger et al. [23] that high mortality rates of *Nitrospinia* could be explained by viral predation.

## Supplementary Figures

| 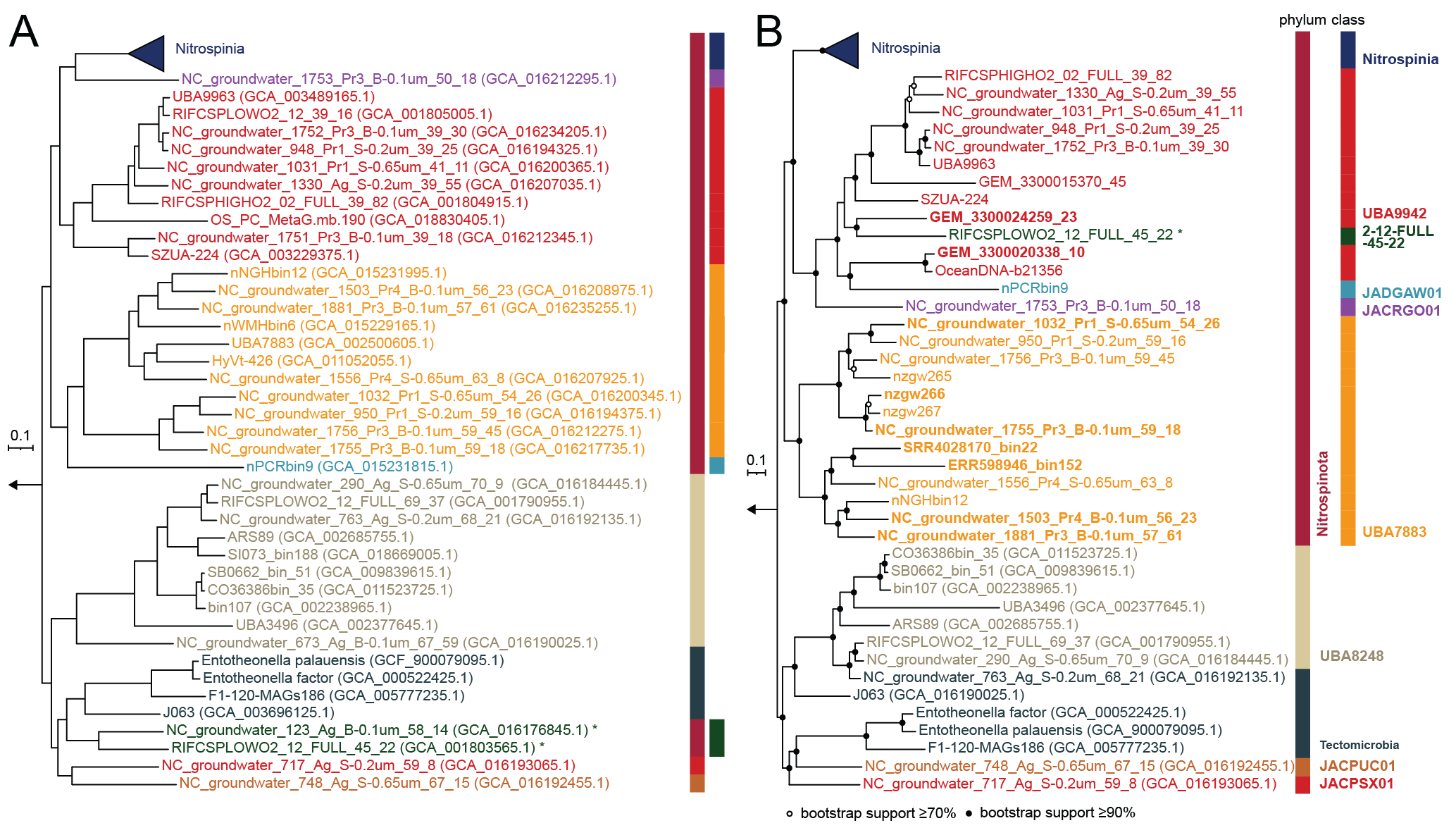 |
| --- |
| **Figure S1**. **Phylogenomic tree of *Nitrospinota* and related phyla.** **A.** Part of the bacterial GTDB FastTree (r207), with the genomes belonging to the phylum Nitrospinota_B (marked with an asterisk) clustering together with the phylum *Ca.* Tectomicrobia. The colored bars next to the tree indicate phylum affiliation, for labels see panel B. **B.** Phylogenomic tree of *Nitrospinota* genomes included in this study dereplicated at ≥ 99% ANI with additional genomes belonging to the phyla UBA8248, Tectomicrobia, JACPUC01, and JACPSX01. Due to the low estimated completeness, the MAG NC_groundwater_123_Ag_B-0.1um_58_14 was excluded during the dereplication step and is thus not included in panel B. The maximum likelihood tree was calculated using IQ-tree with the GTR+F+I+G4 model. Empty circles represent bootstrap support ≥ 70%, black circles represent bootstrap support ≥ 90% of 1000 ultrafast bootstrap replicates. Names from *Nitrospinota* MAGs with > 90% completeness and < 5% redundancy are shown in bold. The colored bars next to the tree show the phylum and class level classification based on GTDB-Tk. See Supplementary Table S2 for genome accession numbers. |

| 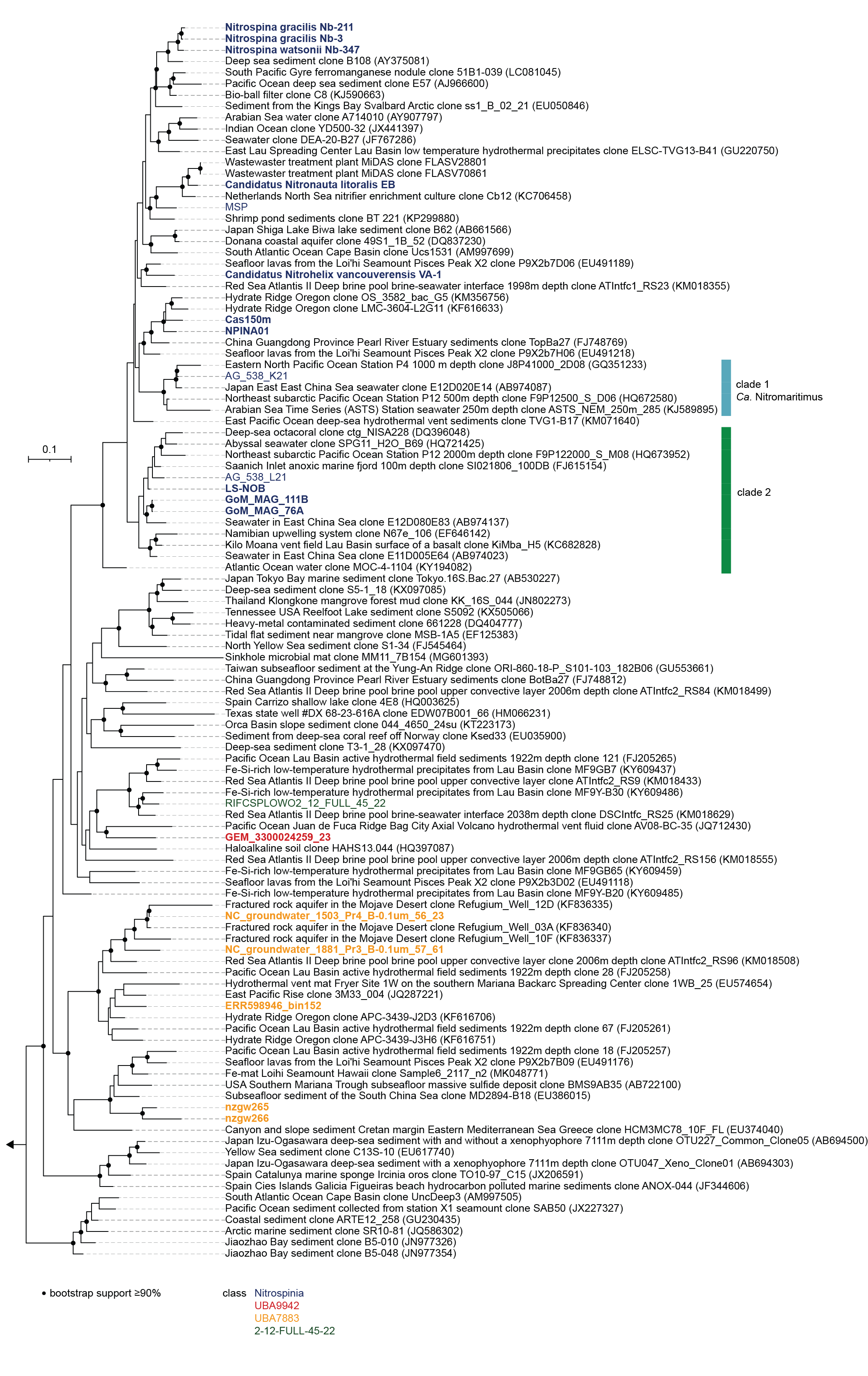 |
| --- |
| **Figure S2.** **Phylogeny of *Nitrospinota*** **16S rRNA gene sequences.** 16S rRNA gene maximum likelihood phylogenetic IQ-tree using near full-length sequences with the SYM+I+G4 model with 1000 ultrafast bootstrap replicates. The black circles represent bootstrap support ≥ 90%. 16S rRNA gene sequences extracted from *Nitrospinota* genomes with > 90% completeness and < 5% redundancy are shown in bold. |

| 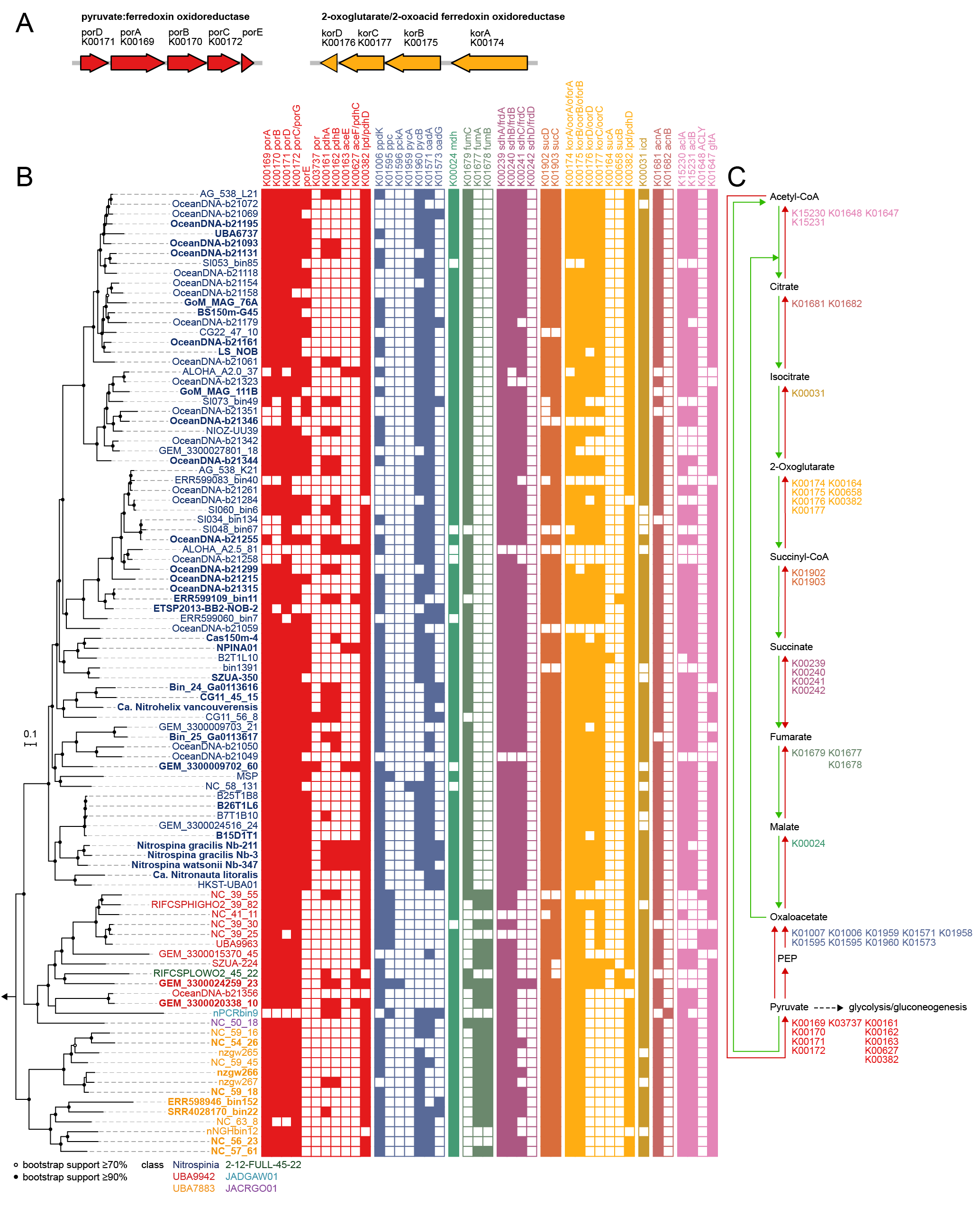 |
| --- |
| **Figure S3. Heatmap showing genes for the reductive and oxidative tricarboxylic acid (TCA) cycle in *Nitrospinota* genomes.** **A.** Schematic illustration of *Nitrospina gracilis* Nb-211 pyruvate:ferredoxin oxidoreductase (porDABCE) and 2-oxoglutarate/2-oxoacid ferredoxin oxidoreductase (korABCD) genes. **B.** On the left, a phylogenomic tree of dereplicated *Nitrospinota* genomes based on concatenated alignments of 92 core protein sequences is shown. The maximum likelihood tree was calculated using IQ-tree with the SYM+I+G4 model. Empty and black circles represent bootstrap support ≥ 70% and ≥ 90%, respectively. Genome names from *Nitrospinota* genomes with > 90% completeness and < 5% redundancy are shown in bold. Some MAG names were shortened, see Supplementary Table S2 for full genome names and accession numbers. **C.** Scheme of the oxidative (green arrows) and reductive (red arrows) TCA cycle. KEGG identification numbers listed next to each other represent alternatives. Abbreviations: K00169, porA pyruvate:ferredoxin oxidoreductase alpha subunit; K00170, porB pyruvate:ferredoxin oxidoreductase beta subunit; K00171, porD pyruvate:ferredoxin oxidoreductase delta subunit; K00172, porC/porG pyruvate:ferredoxin oxidoreductase gamma subunit; porE pyruvate:ferredoxin oxidoreductase epsilon subunit; K03737, por pyruvate-ferredoxin/flavodoxin oxidoreductase; K00161, pdhA pyruvate dehydrogenase E1 component alpha subunit;, K00162, pdhB pyruvate dehydrogenase E1 component beta subunit;, K00163, aceE pyruvate dehydrogenase E1 component;, K00627, aceF/pdhC pyruvate dehydrogenase E2 component (dihydrolipoyllysine-residue acetyltransferase);, K00382, lpd/pdhD dihydrolipoyl dehydrogenase; K01006, ppdK pyruvate, orthophosphate dikinase; K01595, ppc phosphoenolpyruvate carboxylase; K01596, pckA phosphoenolpyruvate carboxykinase (GTP); K01959, pycA pyruvate carboxylase subunit A; K01960, pycB pyruvate carboxylase subunit B; K01571, oadA oxaloacetate decarboxylase (Na+ extruding) subunit alpha; K01573, oadG oxaloacetate decarboxylase (Na+ extruding) subunit gamma; K00024, mdh malate dehydrogenase; K01679, fumC fumarate hydratase class II; K01677, fumA fumarate hydratase subunit alpha; K01678, fumB fumarate hydratase subunit beta; K00239, sdhA/frdA succinate dehydrogenase / fumarate reductase flavoprotein subunit; K00240, sdhB/frdB succinate dehydrogenase / fumarate reductase iron-sulfur subunit; K01902, sucD succinyl-CoA synthetase alpha subunit; K01903, sucC succinyl-CoA synthetase beta subunit; K00174, korA/oorA/oforA 2-oxoglutarate/2-oxoacid ferredoxin oxidoreductase subunit alpha; K00175, korB/oorB/oforB 2-oxoglutarate/2-oxoacid ferredoxin oxidoreductase subunit beta; K00164, sucA 2-oxoglutarate dehydrogenase E1 component; K00658, sucB 2-oxoglutarate dehydrogenase E2 component (dihydrolipoamide succinyltransferase); K00382, lpd/pdhD dihydrolipoamide dehydrogenase; K00031, icd isocitrate dehydrogenase; K01681, acnA aconitate hydratase; K01682, acnB aconitate hydratase 2 / 2-methylisocitrate dehydratase; K15230, aclA ATP-citrate lyase alpha subunit; K15231, aclB ATP-citrate lyase beta subunit; K01648, ACLY ATP citrate (pro-S)-lyase; K01647, gltA citrate synthase |

|  | |
| --- | --- |
| **Figure S4. Heatmap showing key genes in *Nitrospinota* genomes involved in energy metabolism and nitrogen and sulfur assimilation.** On the left the same phylogenetic tree as in Figure S3 is shown. Presence and completeness of marker genes and pathways, respectively, are indicated in the heatmap. | |
| 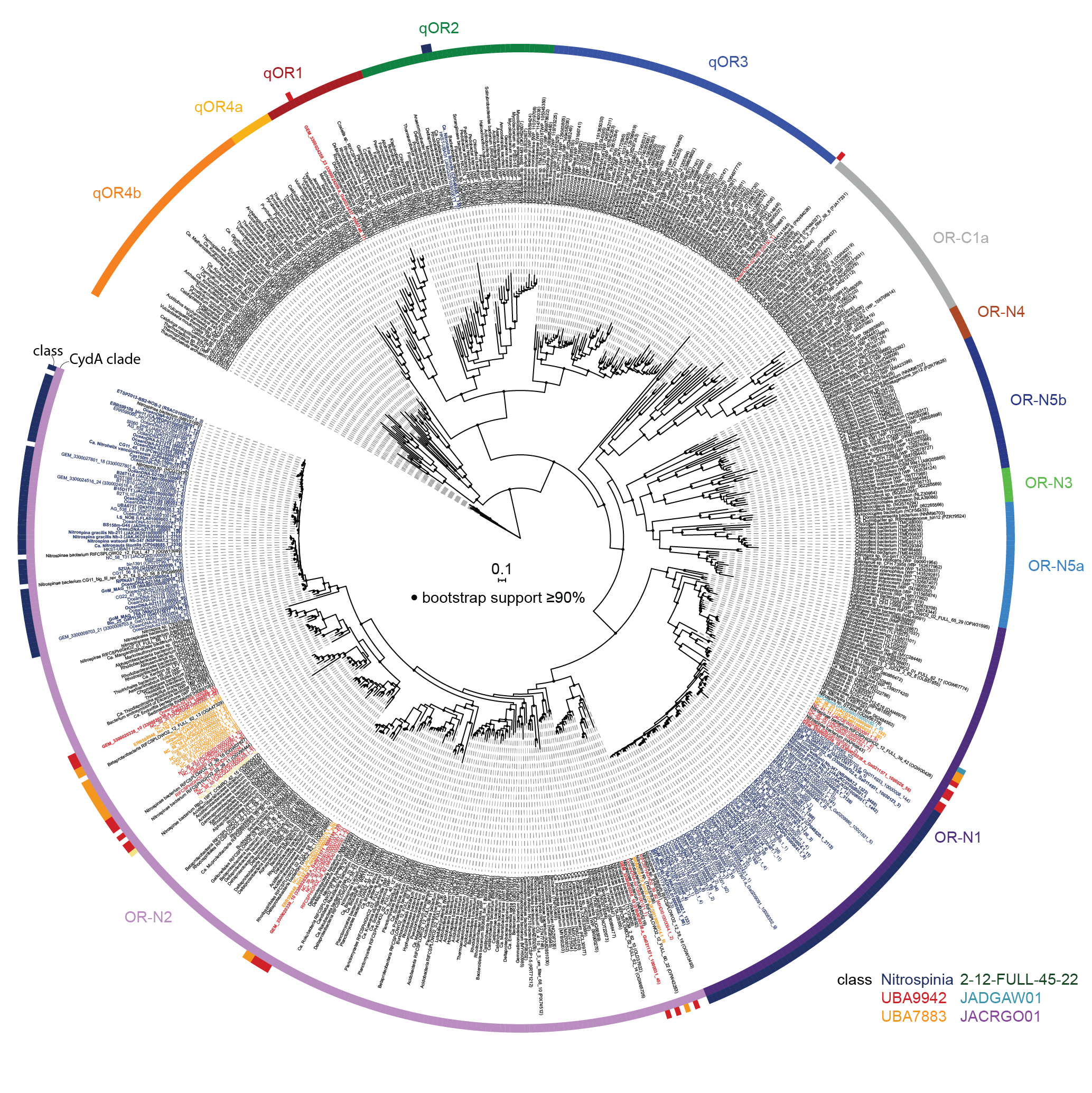 |  |
| **Figure S5. Phylogeny of CydA sequences for quinol-oxidizing *bd*-type oxygen reductases.** The unrooted maximum likelihood tree was calculated using IQ-tree with the VT+F+G4 model. Black circles represent bootstrap support ≥ 90%. The phylogenetic tree was calculated using the CydA sequences of the multiple sequence alignment MSA2 by Murali et al. [24] and CydA clades were labeled accordingly. The outer ring and font color show the *Nitrospinota* class. |  |

| 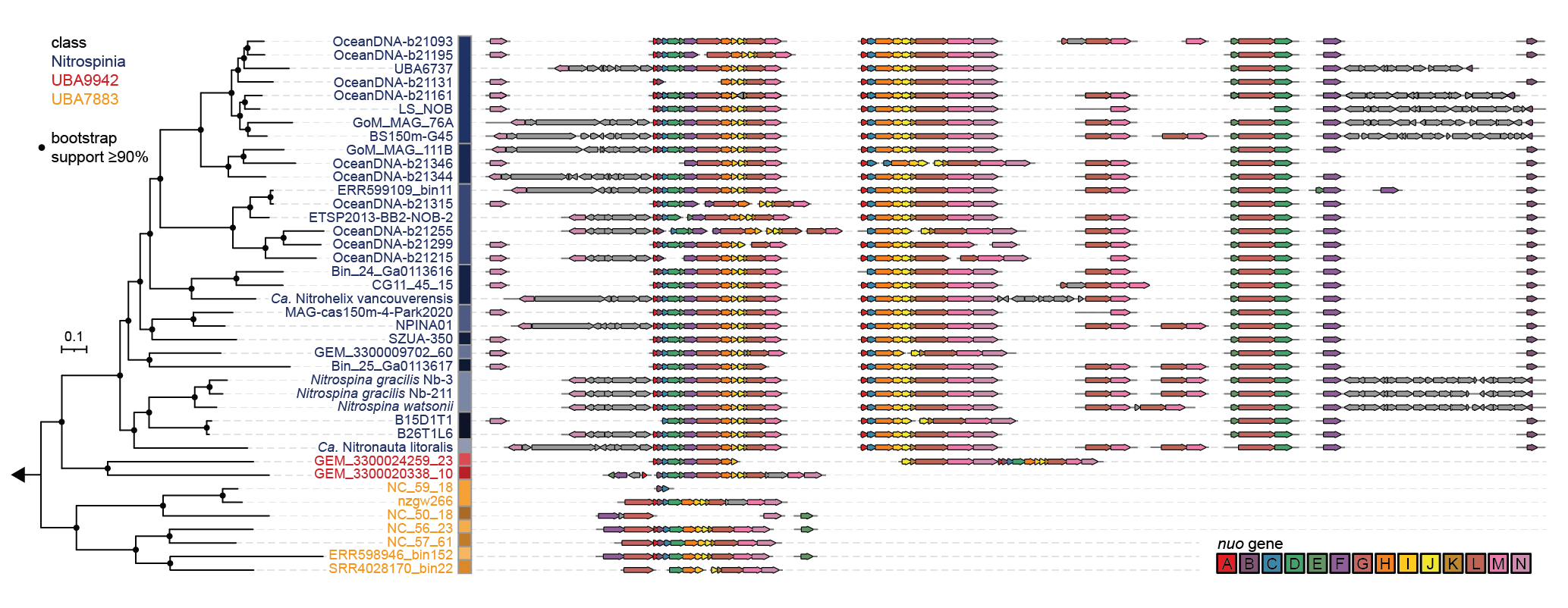 |
| --- |
| **Figure S6.** Schematic illustration of the NADH-quinone dehydrogenase genes (*nuo*). On the left the same phylogenetic tree as in Figure 1 is shown. On the right, an illustration of the *nuo* genes identified in the respective MAGs is shown. Genes for proteins with other functions are shown in grey. Contigs are represented by solid grey lines behind the gene arrows. Arrows are not drawn to scale. |

| 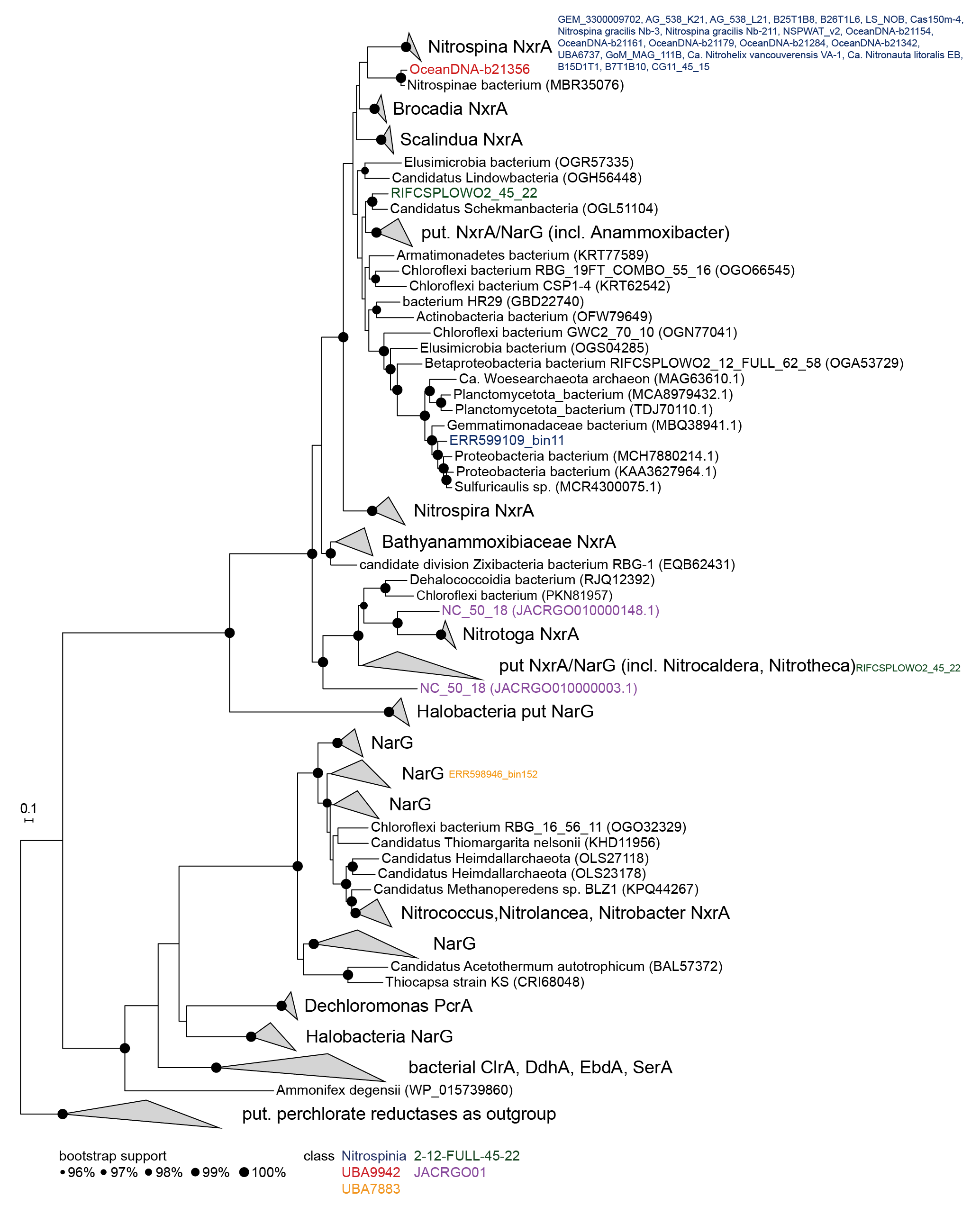 |
| --- |
| **Figure S7. Phylogenetic analysis of selected NxrA and NarG sequences.** Maximum likelihood tree of NxrA, NarG and related proteins calculated using W-IQ-Tree with 1000 ultrafast bootstraps. Only bootstrap support values ≥ 96% are indicated with black circle. The scale bar indicates estimated substitution per amino acid. Selected putative chlorate reductase sequences are used as outgroup. The colors of the *Nitrospinota* sequences indicate their taxonomic affiliation on class level. Abbreviations: Clr, chlorate reductase; Ddh, dimethylsulfide dehydrogenase; Ebd, ethylbenzene dehydrogenase; Nar, nitrate reductases; Nxr, nitrite oxidoreductase; Pcr, perchlorate reductase; Ser, selenate reductase. |

|  |
| --- |
| **Figure S8.** **Phylogeny of the *Nitrospinota* DsrAB enzymes.** The unrooted phylogenetic tree was calculated using a subsampled dataset of DsrAB sequences compiled by Pelikan et al. [25] and labeled accordingly. The maximum likelihood tree was calculated using IQ-tree with 1000 ultra-fast boostrap replicates using the LG+F+G4 model. Black circles represent bootstrap support ≥ 90% of 1000 ultrafast bootstrap replicates. |

|  |
| --- |
| **Figure S9.** **Heatmap showing key genes involved in stress resistance and osmoprotection in *Nitrospinota* genomes.** On the left the same phylogenetic tree as in Figure S3 is shown. Presence and completeness of genes and complexes, respectively, involved in stress resistance and osmoprotection are shown in the heatmap. |

| 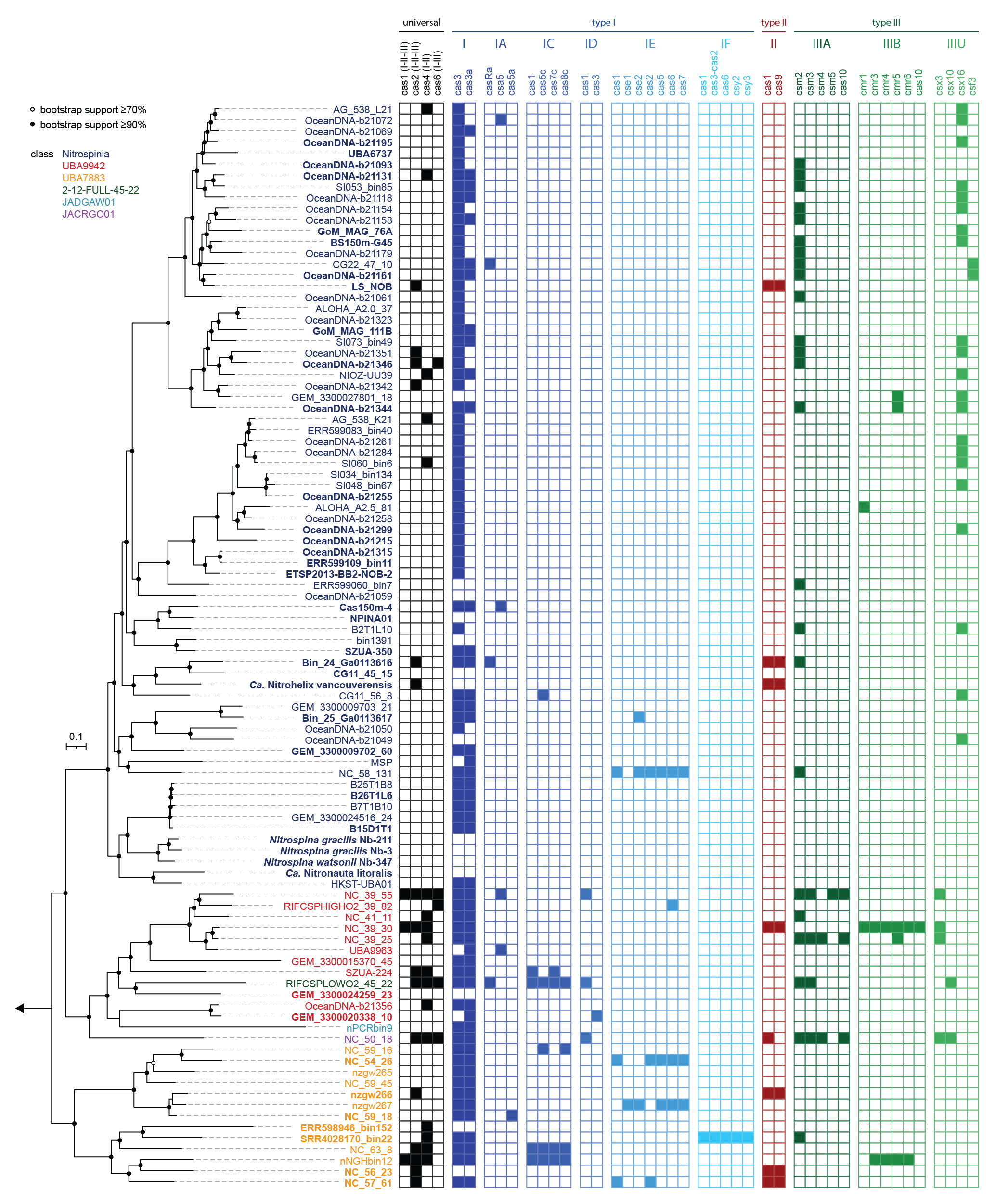 |
| --- |
| **Figure S10. Cas genes identified by CRISPRCasFinder in *Nitrospinota* genomes.** On the left the same phylogenetic tree as in Figure S3 is shown. Presence of Cas genes is shown in the presence/absence matrix. Information on CRISPR arrays identified in *Nitrospinota* genomes can be found in Supplementary Table S7. |

## References

1. Hägerhäll C. Succinate:quinone oxidoreductases variations on a conserved theme. *Biochim Biophys Acta* 1997; **1320**: 107–141.

2. Mlynek G, Sjöblom B, Kostan J, Füreder S, Maixner F, Gysel K, et al. Unexpected diversity of chlorite dismutases: A catalytically efficient dimeric enzyme from *Nitrobacter winogradskyi*. *J Bacteriol* 2011; **193**: 2408–2417.

3. Lees H, Simpson JR. The biochemistry of the nitrifying organisms. Nitrite oxidation by *Nitrobacter*. *Biochem J* 1957; **65**: 297–305.

4. Barnum TP, Coates JD. Chlorine redox chemistry is widespread in microbiology. *ISME J* 2022; 2021.12.08.471835.

5. Maixner F, Wagner M, Lücker S, Pelletier E, Schmitz-Esser S, Hace K, et al. Environmental genomics reveals a functional chlorite dismutase in the nitrite-oxidizing bacterium ‘*Candidatus* Nitrospira defluvii’. *Environ Microbiol* 2008; **10**: 3043–3056.

6. Ngugi DK, Blom J, Stepanauskas R, Stingl U. Diversification and niche adaptations of *Nitrospina*-like bacteria in the polyextreme interfaces of Red Sea brines. *ISME J* 2015; **10**: 1–17.

7. Watson SW, Waterbury JB. Characteristics of two marine nitrite oxidizing bacteria, *Nitrospina gracilis nov. gen. nov. sp.* and *Nitrococcus mobilis nov. gen. nov. sp.* *Arch Microbiol* 1971; **77**: 203–204.

8. Spieck E, Keuter S, Wenzel T, Bock E, Ludwig W. Characterization of a new marine nitrite oxidizing bacterium, *Nitrospina watsonii* sp. nov., a member of the newly proposed phylum *“Nitrospinae”*. *Syst Appl Microbiol* 2014; **37**: 170–176.

9. Uzun M, Alekseeva L, Krutkina M, Koziaeva V, Grouzdev D. Unravelling the diversity of magnetotactic bacteria through analysis of open genomic databases. *Sci Data* 2020; **7**: 1–13.

10. Bazylinski DA, Frankel RB. Magnetosome formation in prokaryotes. *Nat Rev Microbiol* 2004; **2**: 217–230.

11. Lücker S, Nowka B, Rattei T, Spieck E, Daims H. The genome of *Nitrospina gracilis* illuminates the metabolism and evolution of the major marine nitrite oxidizer. *Front Microbiol* 2013; **4**: 1–19.

12. Mueller AJ, Jung MY, Strachan CR, Herbold CW, Kirkegaard RH, Wagner M, et al. Genomic and kinetic analysis of novel Nitrospinae enriched by cell sorting. *ISME J* 2020.

13. Park S-J, Andrei A-Ş, Bulzu P-A, Kavagutti VS, Ghai R, Mosier AC. Expanded Diversity and Metabolic Versatility of Marine Nitrite-Oxidizing Bacteria Revealed by Cultivation- and Genomics-Based Approaches. *Appl Environ Microbiol* 2020; **86**: 1–17.

14. Arenas FA, Díaz WA, Leal CA, Pérez-Donoso JM, Imlay JA, Vásquez CC. The *Escherichia coli btuE* gene, encodes a glutathione peroxidase that is induced under oxidative stress conditions. *Biochem Biophys Res Commun* 2010; **398**: 690–694.

15. Figueiredo MCO, Lobo SAL, Carita JN, Nobre LS, Saraiva LM. Bacterioferritin protects the anaerobe *Desulfovibrio vulgaris* Hildenborough against oxygen. *Anaerobe* 2012; **18**: 454–458.

16. Cabreiro F, Picot CR, Friguet B, Petropoulos I. Methionine Sulfoxide Reductases: Relevance to Aging and Protection against Oxidative Stress. *Ann N Y Acad Sci* 2006; **1067**: 37–44.

17. Burgsdorf I, Sizikov S, Squatrito V, Britstein M, Slaby BM, Cerrano C, et al. Lineage-specific energy and carbon metabolism of sponge symbionts and contributions to the host carbon pool. *ISME J* 2021.

18. Lamborg CH, Hammerschmidt CR, Bowman KL, Swarr GJ, Munson KM, Ohnemus DC, et al. A global ocean inventory of anthropogenic mercury based on water column measurements. *Nature* 2014; **512**: 65–68.

19. Gionfriddo CM, Tate MT, Wick RR, Schultz MB, Zemla A, Thelen MP, et al. Microbial mercury methylation in Antarctic sea ice. *Nat Microbiol* 2016; **1**: 16127.

20. Tada Y, Marumoto K, Takeuchi A. *Nitrospina*-like Bacteria Are Dominant Potential Mercury Methylators in Both the Oyashio and Kuroshio Regions of the Western North Pacific. *Microbiol Spectr* 2021; **9**: 1–11.

21. Starr LD, McCarthy MJ, Hammerschmidt CR, Subramaniam A, Despins MC, Montoya JP, et al. Mercury methylation linked to nitrification in the tropical North Atlantic Ocean. *Mar Chem* 2022; **247**: 104174.

22. Villar E, Cabrol L, Heimbürger‐Boavida L. Widespread microbial mercury methylation genes in the global ocean. *Environ Microbiol Rep* 2020; **12**: 277–287.

23. Kitzinger K, Marchant HK, Bristow LA, Herbold CW, Padilla CC, Kidane AT, et al. Single cell analyses reveal contrasting life strategies of the two main nitrifiers in the ocean. *Nat Commun* 2020; **11**: 767.

24. Murali R, Gennis RB, Hemp J. Evolution of the cytochrome *bd* oxygen reductase superfamily and the function of CydAA’ in Archaea. *ISME J* 2021; **15**: 3534–3548.

25. Pelikan C, Jaussi M, Wasmund K, Seidenkrantz M-S, Pearce C, Kuzyk ZZA, et al. Glacial Runoff Promotes Deep Burial of Sulfur Cycling-Associated Microorganisms in Marine Sediments. *Front Microbiol* 2019; **10**: 1–17.
